# Dendritic processing of spontaneous neuronal sequences for single-trial learning

**DOI:** 10.1038/s41598-018-33513-9

**Published:** 2018-10-11

**Authors:** Tatsuya Haga, Tomoki Fukai

**Affiliations:** RIKEN Center for Brain Science, Hirosawa 2-1, Wako, Saitama, 351-0198 Japan

## Abstract

Spontaneous firing sequences are ubiquitous in cortical networks, but their roles in cellular and network-level computations remain unexplored. In the hippocampus, such sequences, conventionally called preplay, have been hypothesized to participate in learning and memory. Here, we present a computational model for encoding input sequence patterns into internal network states based on the propagation of preplay sequences in recurrent neuronal networks. The model instantiates two synaptic pathways in cortical neurons, one for proximal dendrite-somatic interactions to generate intrinsic preplay sequences and the other for distal dendritic processing of extrinsic signals. The core dendritic computation is the maximization of matching between patterned activities in the two compartments through nonlinear spike generation. The model performs robust single-trial learning with long-term stability and independence that are modulated by the plasticity of dendrite-targeted inhibition. Our results demonstrate that dendritic computation enables somatic spontaneous firing sequences to act as templates for rapid and stable memory formation.

## Introduction

Fast and robust learning is a fundamental ability of the brain and has been extensively explored in the hippocampus and sensory cortices. During spatial navigation, hippocampal place cells rapidly acquire their spatial receptive fields during the first exposure to the spatial environment^1^. In addition to the learning speed, hippocampus can form distinct spatial representations for multiple spatial experiences without interference^2,3^, avoiding overwriting of previous memories. Hippocampal place cells exhibit firing sequences during locomotion^4,5^ and these sequences are replayed during awake and sleep states^6,7^. These replay sequences are widely thought to be crucial for the memory encoding and consolidation processes^5,7,8^, but the circuit mechanisms of rapid and robust memory formation remain largely unknown.

It was recently suggested that a large fraction of place-cell sequences emerge from firing sequences that are ‘pre-played’ in spontaneous activity prior to spatial experience^9,10^. Although the role of spontaneous firing in place-field formation remains controversial^11^, supportive evidence is also accumulating^12,13^. Active roles of spontaneous firing sequences were also reported in the rodent auditory and somatosensory cortices^14^, in which repeated application of sequential stimuli consolidated spontaneous firing sequences existing prior to the sensory experiences. These results suggest that cortical networks have an innate structure to utilize spontaneous firing patterns for sequence learning. In addition, computational models suggest that the lognormal neuronal connectivity observed in the hippocampus^15,16^ and neocortex^17,18^ embeds a rich repertoire of firing sequences into asynchronous irregular states of recurrent neuronal networks^19,20^.

Because the area CA3 is the likely source of preplay sequences observed in the area CA1^21,22^, we build a recurrent network model to demonstrate how spontaneous firing of CA3 neurons enable sequence learning from one-time experience. Unlike the previous models of hippocampal sequence learning^23–27^, our model does not require the preconfigured place fields of individual neurons prior to sequence learning. However, our model assumes that spontaneous firing sequences exist prior to spatial experiences. Our primary interest is whether cortical circuits can rapidly and robustly associate sequences of sensory input patterns with specific preplay sequences. Our model proposes that dendritic coincidence detection is the key of this association. In the hippocampus, the perforant path from the entorhinal cortex (EC) conveys sensory information to the distal dendrites of CA3 pyramidal cells, while recurrent synapses primarily contact their proximal dendrites^28^. Similarly, in neocortical pyramidal cells distal dendrites receive top-down input from the higher cortical areas whereas bottom-up input from the lower cortical areas (or thalamic nuclei) terminates on the proximal dendrites^29^. We suggest that this bipartite architecture of cortical networks allows pyramidal cells to amplify and potentiate coincidence between two input streams.

To show this, we construct a mathematically tractable, yet biologically plausible two-compartment neuron model, which includes Hebbian plasticity in each compartment^30^ and coincidence detection between the compartments. The dendrite of our neuron model performs an operation similar to canonical correlation analysis (CCA) in signal processing^31,32^. Coincidence detection has been demonstrated in the dendrites of neocortical^29,33,34^ and CA1^35,36^ pyramidal cells, but not directly in CA3 pyramidal cells. However, recent experiments have clarified that NMDA spikes are critical determinant of long-term potentiation (LTP) at CA3-to-CA3 synapses and that a burst of a few back-propagating action potentials is sufficient to generate NMDA spikes^37^. Although whether LTP at EC-to-CA3 synapses obeys a similar mechanism is unknown, we hypothesize that CA3 pyramidal cells also perform coincidence detection between distal and proximal dendritic inputs. We propose that the combination of dendritic computation and spontaneous firing sequences promotes the rapid formation of stable place fields and place-cell sequences.

## Results

### Two-compartment neuron model with nonlinear dendritic computation

Dendritic coincidence detection and consequent synaptic plasticity play a pivotal role in the spatial memory encoding modeled below. Based on this principle, we constructed a mathematically tractable neuron model keeping its biological plausibility. We considered a two-compartment neuron model with a somatic compartment describing, in reality, the combination of a soma, basal and proximal dendrites, and a distal dendritic compartment representing the apical tuft dendrite. Assuming that the conductance between two compartments is small^29^, we modeled the activation of each compartment independently as

1 $$x(t)={\rm{f}}(\sum _{j}{w}_{j}^{{\rm{som}}}{I}_{j}^{{\rm{som}}}(t)+\beta y(t-{\rm{\Delta }}t)),$$

2 $$y(t)={\rm{f}}(\sum _{j}{w}_{j}^{{\rm{dnd}}}{I}_{j}^{{\rm{dnd}}}(t)+\beta x(t-{\rm{\Delta }}t)),$$

where *x*(*t*) determines the firing rate of sodium spikes in the somatic compartment, *y*(*t*) is the local activity in the distal dendritic compartment, ${w}_{j}^{{\rm{som}}}$ and ${w}_{j}^{{\rm{dnd}}}$ are synaptic weights on the somatic and dendritic compartments, respectively. The terms *βy*(*t* − Δ*t*) and *βx*(*t* − Δ*t*) represent the threshold modifications of somatic and dendritic spikes by the other compartments^29,33^ and short delay Δ*t* = 1 ms. Unweighted postsynaptic currents ${I}_{j}^{{\rm{som}}}(t)$ and ${I}_{j}^{{\rm{dnd}}}(t)$ generated by each synapse are calculated from the activity ${u}_{j}^{X}(t)$ of presynaptic neuron *j* as

3 $$\frac{d}{dt}{I}_{j}^{X}(t)=-\,\frac{1}{{\tau }_{{\rm{L}}}}{I}_{j}^{X}(t)+{u}_{j}^{X}(t),\,(X={\rm{som}},{\rm{dnd}})$$

where the decay constant of postsynaptic currents *τ*_L_ = 10 ms. Each neuron has a sigmoidal nonlinear response function ${\rm{f}}(I)=1/(1+\exp (\,-\,(I-{\theta }_{{\rm{f}}})))\,\,$with *θ*_f_ = 5 being a constant threshold.

Synchronous activation of the two compartments represented by the product *x*(*t*)*y*(*t*) triggers the dendritic mechanism of coincidence detection, such as calcium spikes in neocortical^29,33^ and CA1^35,36^ pyramidal neurons, which in turn enhances neuronal firing. Thus, the net output firing rate of the two-compartment neuron is expressed as

4 $$z(t)=(1+\gamma y(t))\varphi x(t),$$

where *ϕ* is the maximum firing rate elicitable by local inputs to the somatic compartment, and *γ* is the amplification factor of calcium spikes, and *z*(*t*) gives the output firing rate of the two-compartment neuron. The above equation takes into account the experimental observations that activation of distal dendrites increases the gain of somatic firing rate^29,33^.

We express the learning rule for the two-compartment neuron as

5 $${\rm{\Delta }}{w}_{j}^{{\rm{som}}}(t)=\eta [(1-\alpha )x(t)(x(t)-{\theta }^{{\rm{som}}})+\alpha x(t)y(t)](1-x(t)){I}_{j}^{{\rm{som}}}(t),$$

6 $${\rm{\Delta }}{w}_{j}^{{\rm{dnd}}}(t)=\eta [(1-\alpha )y(t)(y(t)-{\theta }^{{\rm{dnd}}})+\alpha x(t)y(t)](1-y(t)){I}_{j}^{{\rm{dnd}}}(t),$$

where *α* is a constant that determines the relative magnitude of the potentiation caused by calcium spikes. In both equations, the first terms in brackets represent Hebbian synaptic plasticity induced by local activities *x*(*t*) and *y*(*t*) by BCM theory^38,39^. While BCM theory was originally introduced to describe the relationship between somatic firing rate and weight changes, a similar rule to BCM theory was also shown for dendritic activity^40^. The second terms in brackets express the LTP effect generated by coincident proximal and distal dendritic inputs, as observed in CA1 pyramidal neurons^35^ and neocortical neurons^41^. Overall factors (1 − *x*(*t*)) and (1 − *y*(*t*)), which do not change the direction of weight changes, were multiplied to match to the objective function we present later. As in the original BCM theory, moving thresholds *θ*^som^(*t*) = *c*_0_E[*x*(*t*)]^2^, *θ*^dnd^(*t*) = *c*_0_E[*y*(*t*)]^2^ prevent run-away evolution of synaptic strength.

### PCA-like and CCA-like learning in the two-compartment neuron model

It is worth noting that the present learning rule for two-compartment neurons (*β* = 0) is derived from the following objective function:

7 $$\begin{array}{rcl}L & = & (1-\alpha )(\frac{1}{2}{\rm{E}}[x{(t)}^{2}]+\frac{1}{2}{\rm{E}}[y{(t)}^{2}]-{c}_{0}{\rm{E}}{[x(t)]}^{3}-{c}_{0}{\rm{E}}{[y(t)]}^{3})\\  &  & +\alpha {\rm{E}}[x(t)y(t)],\end{array}$$

by gradient ascent

8 $${\rm{\Delta }}{w}_{j}^{X}=\eta \frac{dL}{d{w}_{j}^{X}}\,(X={\rm{som}},{\rm{dnd}}).$$

This objective function implies the maximization of second-order moments E[*x*(*t*)^2^], E[*y*(*t*)^2^] and correlation E[*x*(*t*)*y*(*t*)] in conjunction with the minimization of means E[*x*(*t*)]^3^ and E[*y*(*t*)]^3^. Therefore, the learning rule achieves the combination of PCA-like^30^ and CCA-like^31,32^ learning of input vectors ${{\bf{I}}}^{X}(t)=(\ldots ,{I}_{j}^{X}(t),\ldots )\,(X={\rm{som}},{\rm{dnd}})$ under a homeostatic constraint, where *α* determines the relative weight of CCA. In this paper, single-compartment neurons have only somatic compartment and hence perform only PCA-like learning supposed by BCM theory. In contrast, two-compartment neurons perform dual learning, that is, PCA-like learning within each compartment and CCA-like learning between the two compartments.

**Figure 1:**
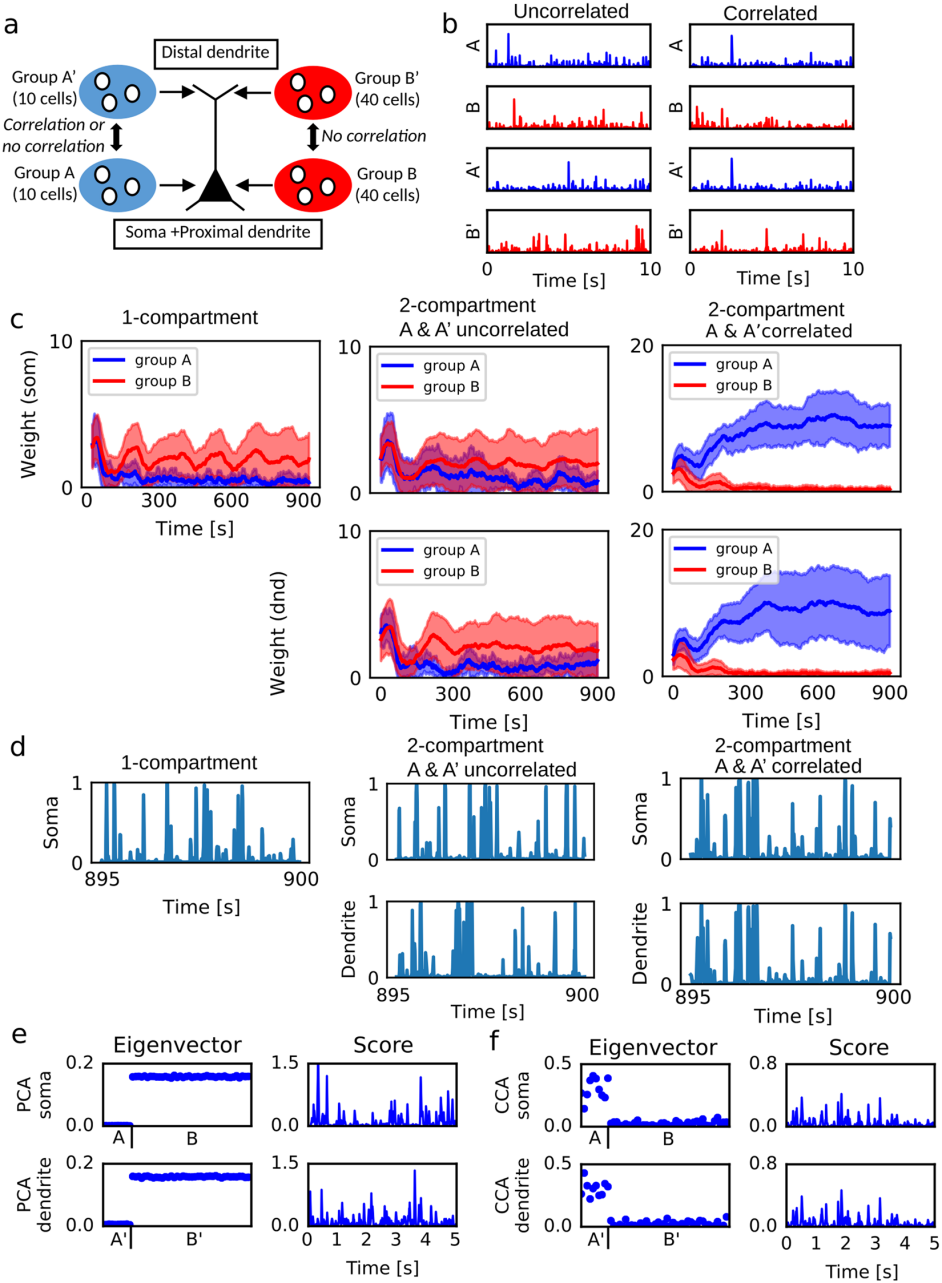
PCA- and CCA-like learning in a two-compartment neuron model. (**a**) Simulation settings. Each compartment receives inputs from 50 neurons. Each group A or A’ consists of 10 input neurons, and each group B or B’ of 40 neurons. (**b**) An example of input neuron activities when the groups A and A’ were correlated or uncorrelated. (**c**) Time evolution of synaptic weights are shown for the single-compartment model (left) and the two-compartmental model receiving uncorrelated (center) or correlated (right) inputs from A and A’. The single compartment neuron only received inputs from A and B. The means (lines) and standard deviations (shaded areas) are shown. (**d**) The activities of the neuron models were shown for the same simulation settings as in c. (**e**) PCA were applied to signals simulated in the same setting as in c when A and A’ were correlated. The eigenvectors (left) and the scores of the first PCs (right) are shown. (**f**) CCA were applied to signals simulated in the same setting as in c when A and A’ were correlated.

The learning behavior of the two-compartment neuron significantly varied depending on the correlation pattern of inputs. In Fig. 1a, the somatic and dendritic compartments received synaptic inputs from minority groups (A and A’) and majority groups (B and B’) of input neurons. Activities of these neurons were strongly correlated within each group but were uncorrelated between pairs of groups A-B’, B-A’ and A’-A’. We conducted simulations when A and A’ were either correlated or uncorrelated (Fig. 1b). When groups A and A’ were uncorrelated, synapses from groups B and B’ were potentiated more strongly than those from A and A’ (Fig. 1c, center). Accordingly, the activities of the two compartments were governed by inputs from groups B and B’, and hence were mutually uncorrelated (Fig. 1d, center). In this case, the learning performance was essentially the same as that of the single-compartment model (Fig. 1c, left: *α* = 0, no inter-compartment interaction). By contrast, when the activities of groups A and A’ were correlated, synapses from A and A’ were selectively potentiated whereas those from B and B’ were depressed (Fig. 1c, right). Accordingly, the two compartments exhibited correlated activities after learning (Fig. 1d, right). Note that in the two-compartment model output firing rate was approximately proportional to somatic activity.

For comparison, we calculated the principal components of input vectors **I**^som^(*t*) and **I**^dnd^(*t*) when groups A and A’ were correlated. As expected, the first principal components extracted by PCA in the soma and dendrite were uncorrelated inputs from groups B and B’, respectively, and the scores (signals projected onto PC1 eigenvectors) were also uncorrelated between the two compartments (Fig. 1e). Then the pair of input vectors was analyzed by CCA, which extracted correlated inputs from groups A and A’ and also yielded highly correlated scores (Fig. 1f). Thus, CCA and the two-compartment neuron model operate similarly on correlated somatic and dendritic inputs.

These results imply that CCA-like learning of the two-compartment neuron model can extract a minor input component to one compartment if a coincident input is given to the other. The extraction of weak inputs based on correlation across compartments is a critical difference between our learning rule and conventional Hebbian learning, which basically extracts only major input components. However, if there is no coincident activity between the compartments, each compartment implements independent Hebbian learning and acts like an independent neural unit.

### CCA-like learning requires multiplicative gain modulation

We implemented the following two types of inter-compartment interactions: multiplicative gain modulation and threshold modulation. Our learning rule assumes that the multiplicative soma-dendrite coupling *x*(*t*)*y*(*t*) induces LTP and the objective function suggests that this gives a major contribution to CCA-like learning. However, the parameter dependence of learning behavior and the contribution of threshold modulation remain to be clarified. Therefore, we performed simulations of single cells with different parameter settings. We summarized the results in each setting by calculating differences in synaptic weights from input group A (A’) and group B (B’) on the two compartments:

9 $$\sum _{j\in {\rm{group}}\,{\rm{A}}}{w}_{j}^{{\rm{som}}}-\sum _{j\in {\rm{group}}\,{\rm{B}}}{w}_{j}^{{\rm{som}}},\sum _{j\in {\rm{group}}\,{\rm{A}}^{\prime} }{w}_{j}^{{\rm{dnd}}}-\sum _{j\in {\rm{group}}\,{\rm{B}}^{\prime} }{w}_{j}^{{\rm{dnd}}}.$$

Positive values mean the dominance of CCA-like learning, and negative values mean the dominance of PCA-like learning.

**Figure 2:**
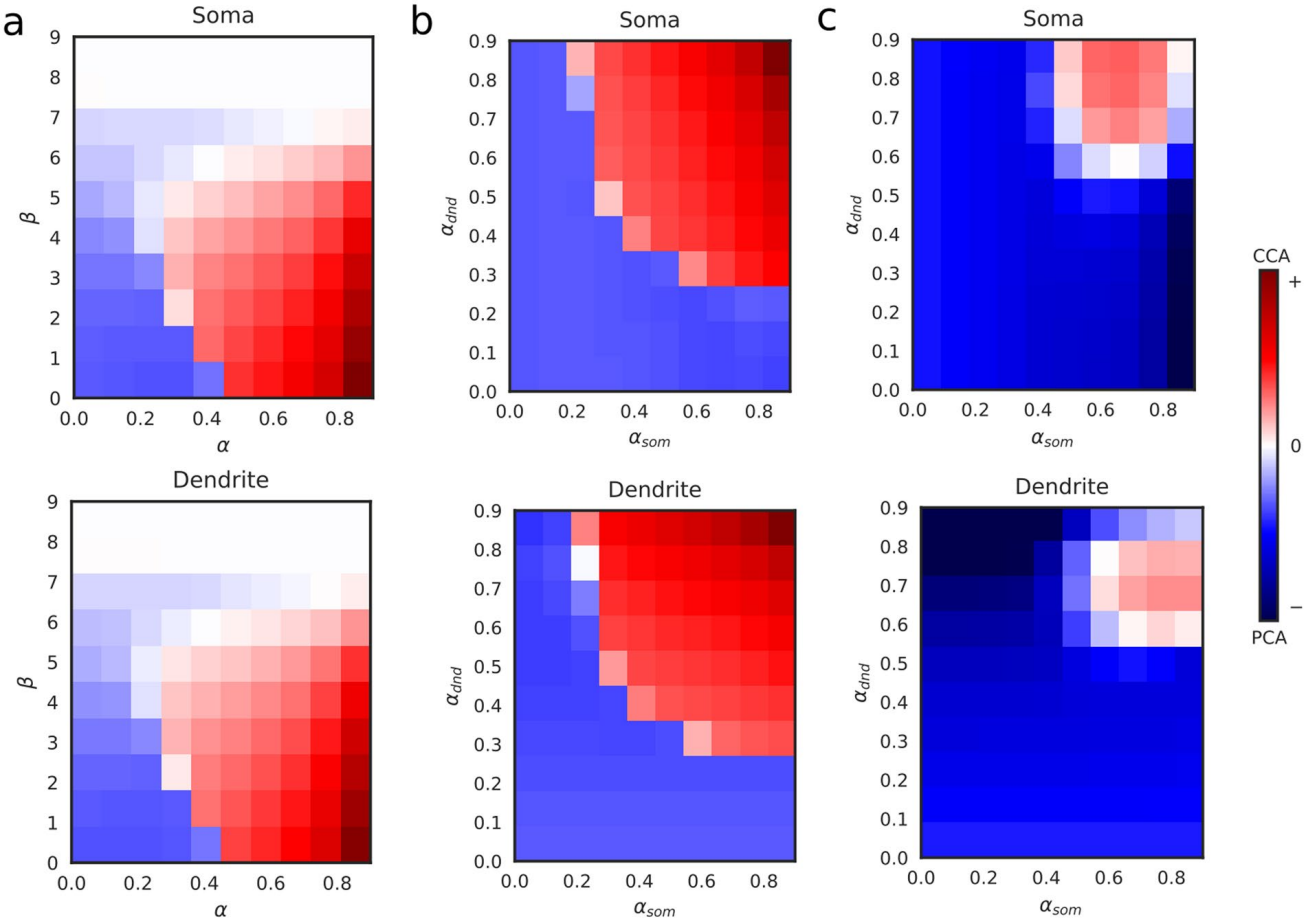
CCA-learning depends on multiplicative gain modulation. (**a**) Learning behavior of the soma (top) and dendrite (bottom) was evaluated for various values of *α* (multiplicative soma-dendrite coupling) and *β* (threshold modulation). Red and blue colors refer to CCA-like learning and PCA-like learning, respectively. (**b**) Learning behavior was evaluated at *β* = 0 for different values of *α* in the soma (*α*_som_) and dendrite (*α*_dnd_). (**c**) Learning behavior was evaluated for the plasticity rules given by Eqs (10) and (11) (BCM theory in which synaptic weights were modified by the product of presynaptic and postsynaptic activities).

First, we checked how the learning behavior depends on *α* (the relative strength of the multiplicative soma-dendrite coupling in learning rule) and *β* (threshold modulation). As shown in Fig. 2a, CCA-like learning is observed mainly in the region *α* > 0.5. This threshold for *α* is decreased by increasing the value of *β*. However, CCA-like learning did not appear for *α* = 0, and all synaptic weights approximately vanished because of homeostasis when *β* was too high (white regions in Fig. 2a). Second, we fixed *β* at zero and set different values to *α* in learning rules for ${w}_{j}^{{\rm{som}}}$ (*α*_som_) and ${w}_{j}^{{\rm{dnd}}}$ (*α*_dnd_), by which we confirmed that the contribution of the multiplicative coupling have to be high in both compartments for CCA-like learning (Fig. 2b). Third, we relaxed the assumption that the soma-dendrite coupling always cause LTP: we defined neural activities (or calcium influx) in the two compartments as *z*_som_(*t*) = (1 − *α*_som_)*x*(*t*) + *α*_som_*x*(*t*)*y*(*t*) and *z*_dnd_(*t*) = (1 − *α*_dnd_)*y*(*t*) + *α*_dnd_*x*(*t*)*y*(*t*), and used BCM learning rules

10 $${\rm{\Delta }}{w}_{j}^{{\rm{som}}}(t)=\eta {z}_{{\rm{som}}}(t)({z}_{{\rm{som}}}(t)-{\theta }^{{\rm{som}}})(1-x(t)){I}_{j}^{{\rm{som}}}(t),$$

11 $${\rm{\Delta }}{w}_{j}^{{\rm{dnd}}}(t)=\eta {z}_{{\rm{dnd}}}(t)({z}_{{\rm{dnd}}}(t)-{\theta }^{{\rm{dnd}}})(1-y(t)){I}_{j}^{{\rm{dnd}}}(t).$$

We also calculated the values of sliding thresholds *θ*^som^ and *θ*^dnd^ using *z*_som_(*t*) and *z*_dnd_(*t*), respectively. Multiplicative amplification between somatic and dendritic activity and BCM-like learning rules are based on experiments and detailed simulation studies as we mentioned before. Due to the threshold parameters in Eqs 10 and 11, coincident input to the somatic and dendritic compartments do not necessarily induce LTP and there is no theoretical constraint for CCA. Even in this case, we observed CCA-like learning for high *α* values (Fig. 2c). These results suggest that strong multiplicative gain amplification in both compartments is necessary and sufficient for CCA-like learning, but threshold modulation alone is not sufficient for it. However, we will show later that threshold modulation is required for the learning performance of the network model.

### The role of inhibitory feedback in the two-compartment neuron model

**Figure 3:**
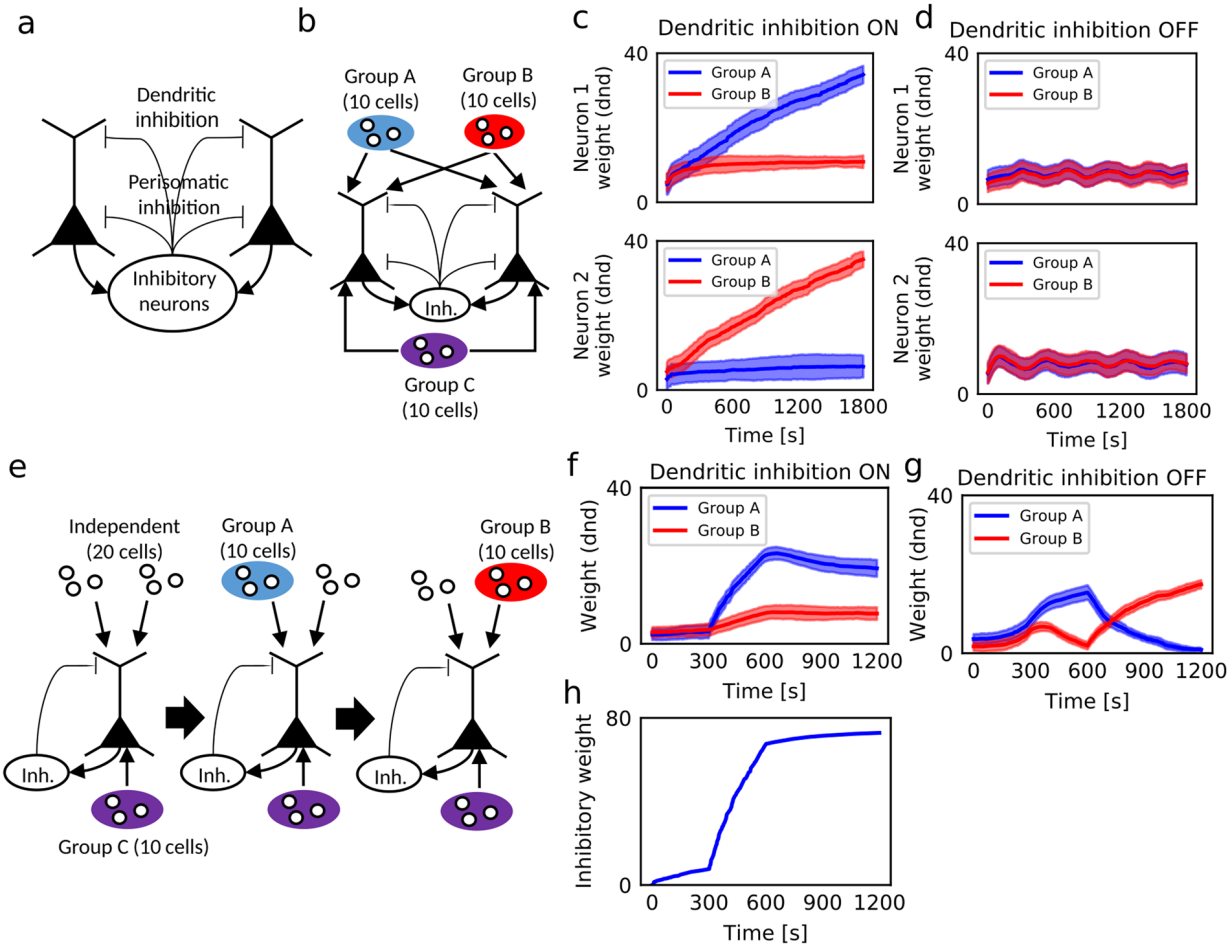
Roles of plastic inhibitory feedback to the distal dendritic compartment. (**a**) Inhibitory feedback model is schematically illustrated. Inhibitory interneurons project to both somatic and dendritic compartments. (**b**) In this simulation setting, two pyramidal neurons projected to an inhibitory neuron and received inhibitory feedback at the somatic and dendritic compartments. In addition, pyramidal neurons received common somatic inputs from excitatory cell group C and mixed dendritic inputs from two mutually-uncorrelated excitatory cell groups A and B. The activity of cell group C was correlated with the activities of cell groups A and B with equal magnitudes. (**c**,**d**) Time evolution of synaptic weights on the dendritic compartments of the two cells with (**c**) or without (**d**) dendritic inhibition. The means (lines) and standard deviations (shaded areas) of synaptic weights are shown. (**e**) A single pyramidal neuron with inhibition fed back onto its dendrite received somatic inputs from a cell group C and dendritic inputs from two cell groups A and B. Activities of input neurons in groups A and B were initially uncorrelated within each group and with other groups. At time 300 sec, correlations were introduced within group A and between groups A and C. At time 600 sec, neurons in group A returned to an uncorrelated state, but neurons in group B became correlated within the group and with group C. (**f**,**g**) Time evolution of excitatory synaptic weights on the dendritic compartment with (**f**) or without (**g**) dendritic inhibition. (**h**) Time evolution of inhibitory synaptic weights on the dendritic compartments is displayed.

The hippocampus has two major types of interneurons, one serving perisomatic inhibition and the other serving dendritic inhibition^42,43^. We modeled the effects of these inhibitory feedback projections ${I}_{i}^{{\rm{sominh}}}(t)$ and ${I}_{i}^{{\rm{dndinh}}}(t)$ in the two-compartmental neuron model (Fig. 3a). We determined the output from each inhibitory unit by the random projection of outputs from all pyramidal neurons (see Methods). Pyramidal neuron *i* was modeled as a two-compartment model with inhibitory feedback:

12 $${x}_{i}(t)={\rm{f}}(\sum _{j}{w}_{ij}^{{\rm{som}}}{I}_{j}^{{\rm{som}}}(t)-\sum _{j}{v}_{ij}^{{\rm{som}}}{I}_{j}^{{\rm{sominh}}}(t)+\beta {y}_{i}(t-{\rm{\Delta }}t)),$$

13 $${y}_{i}(t)={\rm{f}}(\sum _{j}{w}_{ij}^{{\rm{dnd}}}{I}_{j}^{{\rm{dnd}}}(t)-\sum _{j}{v}_{ij}^{{\rm{dnd}}}{I}^{{\rm{dndinh}}}(t)+\beta {x}_{i}(t-{\rm{\Delta }}t)),$$

where ${v}_{i}^{{\rm{som}}}$ and ${v}_{i}^{{\rm{dnd}}}$ are inhibitory synaptic weights.

It has been observed experimentally that not only excitatory but also inhibitory synapses exhibit activity-dependent plasticity^44^. Although the property of inhibitory synaptic plasticity has not been fully understood, in this study inhibitory weights for the distal dendritic compartment are modified by a similar learning rule to excitatory synapses:

14 $${\Delta }{v}_{i}^{{\rm{dnd}}}={\eta }^{{\rm{inh}}}((1-\alpha )y(t)(y(t)-{\theta }^{{\rm{inh}}})+\alpha x(t)y(t))(1-y(t)){I}^{{\rm{inh}}}(t).$$

In this expression, *θ*^inh^ is a constant threshold and was fixed at 0.5 throughout this research (we note that the choice of this parameter value is not crucial for the performance of this model).

We clarified the role of the plastic dendritic inhibition in CCA-like learning in two conditions. First, we simulated a network of two pyramidal neurons and an inhibitory neuron population (Fig. 3b). When a somatic input common to the two pyramidal neurons was correlated with dendritic inputs to both neurons with equal magnitudes, these neurons selectively learned different dendritic inputs in the presence of dendritic inhibition (Fig. 3c), but not in its absence (Fig. 3d). Though the present model also had lateral inhibition between the soma and somatic activity was amplified by dendritic activity (Eq. 4), the intersomatic lateral inhibition alone was insufficient for the separate learning of dendritic inputs. Thus, in this model, the functional specialization of dendrites requires dendritic inhibition. In the second case, we examined the robustness of dendritic excitatory synapses against changes in correlation structure of synaptic inputs (Fig. 3e). Without dendritic inhibition, an abrupt change in correlations between somatic and dendritic inputs rapidly eliminated the previously learned dendritic excitatory synapses (Fig. 3g). By contrast, dendritic inhibition prevented the rapid loss of synaptic memory traces (Fig. 3f). This stability was due to the potentiation of dendritic inhibitory synapses during the learning of the initial correlation structure between groups A and C (Fig. 3h). The potentiated dendritic inhibition changed the excitation-inhibition balance of the dendritic compartment such that its responses to the learned input pattern were enhanced whereas those to other input patterns were suppressed.

Thus, in our model the potentiation of dendritic inhibition separates and stabilizes the receptive fields on the dendrites acquired by CCA-like learning. We will show later that these properties play crucial roles in the robust memory encoding in a recurrent network model of the two-compartment neurons.

### Robust single-trial learning of place fields by two-compartment neural network

**Figure 4:**
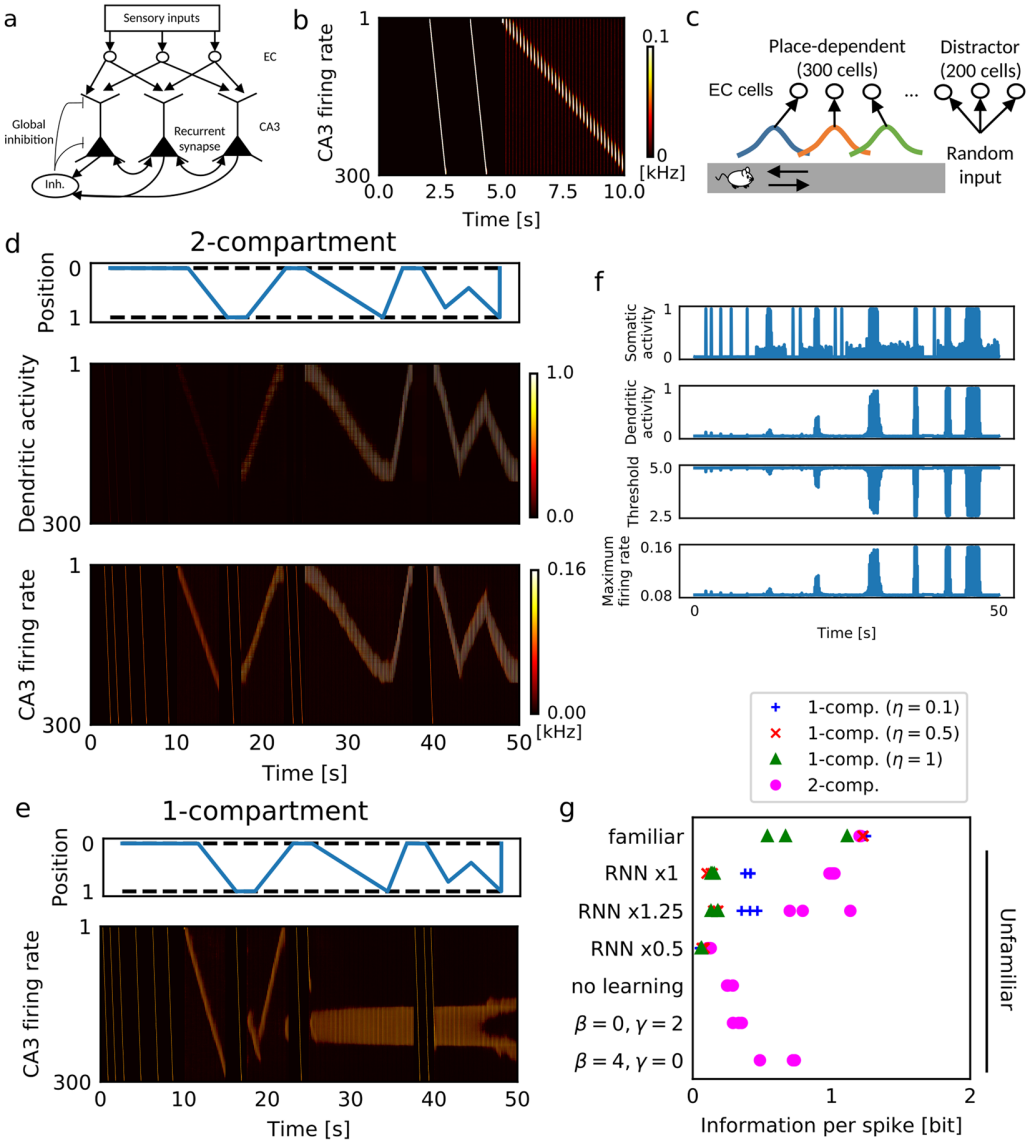
Robust single-trial learning of place fields on a one-dimensional track. (**a**) Our CA3 network model consists of 500 EC neurons projecting to the distal compartments of 300 two-compartment CA3 neurons, which have inhibitory feedback to both distal dendritic and somatic compartments. DG input activates neuron 1 to neuron 10 of CA3 in a probabilistic manner. (**b**) DG-evoked preexisting activity patterns in CA3 were simulated without EC input. The animal was immobile from 0 to 5 sec and ran from 5 to 10 sec. (**c**) The behavioral paradigm and activities of EC neurons in the present simulations. Position-dependent sensory features are encoded by 300 EC neurons, whereas other 200 EC neurons (neuron ID 300 to 500) show position-independent distractor activity. (**d**,**e**) Activities of the two-compartment network model (**d**) and single-compartment network model (**e**) for animal’s movements shown in the top panels. The single-compartment network model was simulated with *η* = 0.5. (**f**) Time evolution during learning is shown for the dynamical variables of the two-compartment neuron. The examples were from CA3 neuron #100. (**g**) Average information per spike was calculated in various conditions. Three simulation trials were performed in each condition with different initial conditions. The strength of recurrent connections was measured relative to the connection strength used in c and d. In simulating familiar tracks, we used the initial weights of EC-to-CA3 synapses optimized to generate place-dependent firing. In the simulations of unfamiliar tracks, these initial weights were randomly shuffled.

Using the two-compartment neuron model and the inhibitory feedback model described above, we constructed a CA3 recurrent network model to investigate the role of dendrites in sequence memory (Fig. 4a). In this model, the somatic compartments of pyramidal neurons receive excitatory recurrent connections, theta-band (7 Hz) oscillatory input from the medium septum^5^ and random noise, while the dendritic compartments receive inputs from the entorhinal cortex (EC). Excitatory connections are reciprocally wired such that the recurrent network can propagate firing sequences^5,45^. We introduced short-term synaptic plasticity which facilitates propagation of sequential activity^45^. During run, we induced theta sequences along recurrent connections by suppression of the decay speed of short-term depression at recurrent connections and externally induced homogenous theta oscillation^5^ (Fig. 4b). Acetylcholine can exert this modulatory effect on neurotransmitter release in the hippocampus^46,47^. During immobility and first run, we induced external triggers in a small portion of CA3 neurons to initiate preexisting firing sequences (Fig. 4b). The dentate gyrus (DG) may deliver this trigger, but noise may also activate spontaneous firing sequences^45^. Here, we used external triggers because the size of our network model was too small to allow arbitrary starting point of learning.

We considered an animal running back and forth on a one-dimensional (1D) track. During a run, position-dependent sensory features on the track activate some EC neurons sequentially, while noise input activates others (distractors) randomly (Fig. 4c). Prior to the first run, there is no way for the animal to know the sensory features and their order of appearance along the track. Therefore, the initial weights of EC-to-CA3 projections were chosen randomly, and accordingly dendritic activity showed no initial place-dependence. The position-dependent EC activity may represent local landmarks in the lateral EC or the firing fields of grid cells in the medial EC^48^.

The animal was initially immobile at an endpoint of an unfamiliar track, where sequences were randomly triggered (Fig. 4d, top), which in turn activated sequential spontaneous CA3 activity (Fig. 4d, bottom). These sequences were initially not associated to any sensory information (hence any spatial information) represented in EC. However, during the first traversal the dendritic compartments rapidly learned to associate sequential EC-to-CA3 inputs with the triggered firing sequence (Fig. 4d, middle). During subsequent runs, in which triggers were no longer provided, dendritic activity established control of somatic activity, and hence of sequence propagation. Thus, without any pre-configured place fields, CA3 neurons showed clear place-dependent firing in the second and third traversals across an unfamiliar track (Fig. 4d). We note that the modulations of the gain and threshold of somatic firing rates promoted the learning of place fields (Fig. 4f).

For comparison, we constructed a single-compartment network model and trained it on the same spatial navigation task. This model received both recurrent synapses and EC inputs at the somatic compartments (thus, the dendritic compartments were passively driven by somatic activity and played no active role). The model failed to form place fields (Fig. 4e). To maintain a similar learning speed, we used a relatively large value of the learning coefficient. However, in this condition noise input (from the distractor EC neurons) easily disturbed the formation of place fields before they became robust. On the other hand, if the learning coefficient was small, firing sequences could not follow changes in the movement directions of the animal at both ends of the maze. Thus, the separation of afferent inputs and recurrent inputs by dendrites is necessary for the efficient use of spontaneous firing sequences in memory formation.

We assessed the quality of the place fields formed in various simulation conditions by means of “information per spike”^49^, a measure based on the mutual information between neural activity of each cell and animal’s position (see Methods). In both models the average mutual information simulated with spatially-structured EC-to-CA3 projections was high in a familiar track (Fig. 4g). However, in an unfamiliar track only the two-compartment model acquired highly place-dependent neural activity, but the single-compartment model exhibited low mutual information for all three values of learning coefficient. The performance of the two-compartment model in the unfamiliar track was also impaired if we turned off the plasticity effect (learning coefficient *η* = 0). Importantly, if we decreased the initial weights of recurrent synapses (by a multiplicative factor of 0.5), the two-compartment model failed to learn the unfamiliar track, indicating the crucial role of spontaneous firing sequences in the place field formation. As expected, increasing the weights (1.25 times) did not degrade the performance in learning. We also checked the importance of gain modulation and threshold modulation in our activity model. Turning off threshold modulation (*β* = 0, γ = 2) strongly impaired the performance, whereas the effect of removing gain modulation (*β* = 4, γ = 0) was modest (Fig. 4g). Thus, the propagation of firing sequences is largely controlled by threshold modulation, whereas gain modulation regulates sequence learning. Both types of inter-compartment interactions are necessary for our network model.

### Learning place fields from activities of grid cells

**Figure 5:**
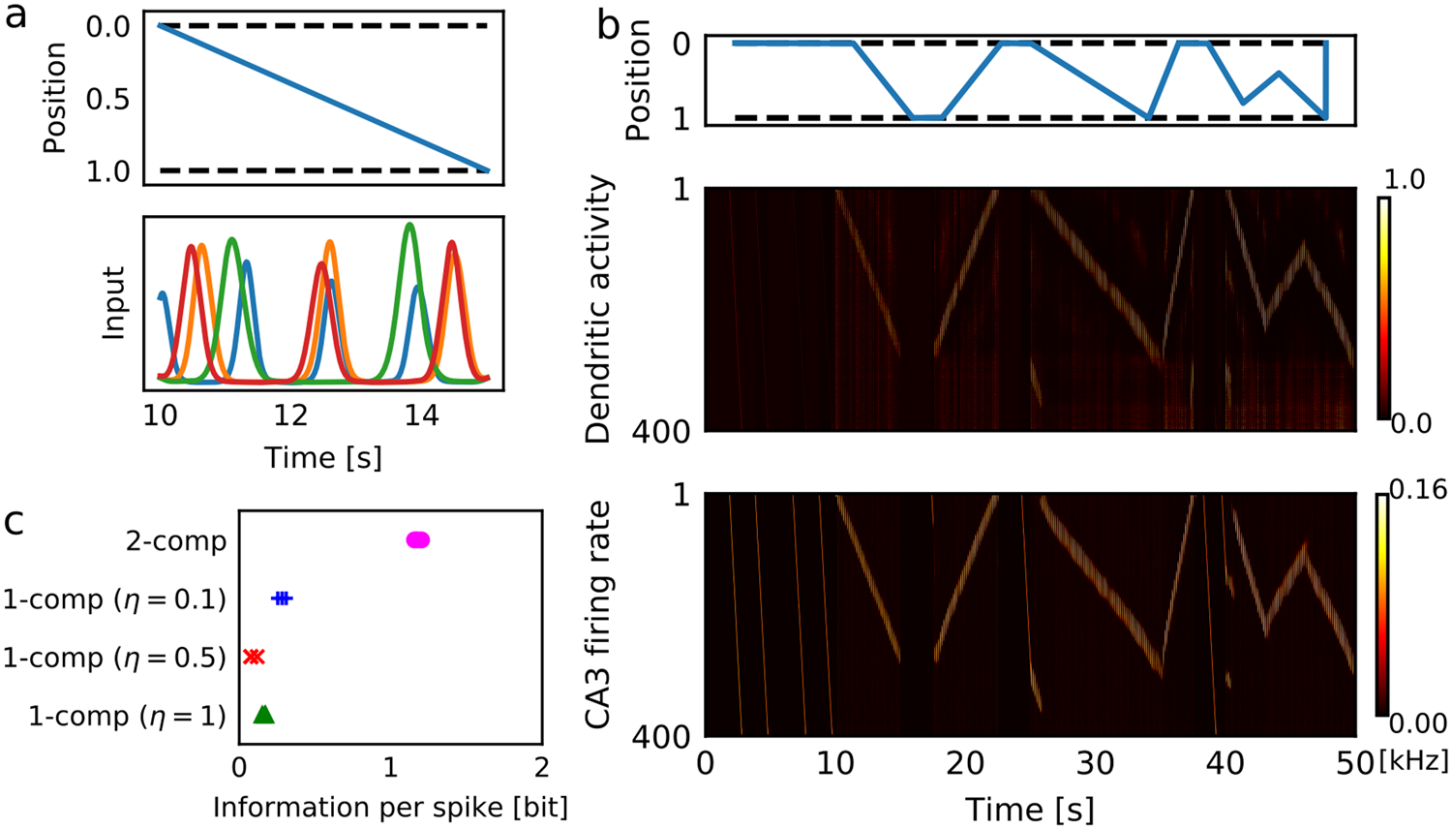
Learning of place fields from activities of grid cells. (**a**) Activity patterns of four EC neurons are shown during a traversal on the 1-D track. Each color shows activity of an EC neuron. The activity traces were smoothed by a Gaussian filter of 100-ms width, which makes the modulations by theta oscillation invisible. (**b**) Activities of the two-compartments are displayed during the animal’s movements shown in the top panel. (**c**) Average information per spike was calculated in various conditions. Three simulation trials were performed in each condition for different realizations of the network and parameters of grid cells.

In the previous section, we showed that our model can rapidly associate sequential input patterns to internal firing sequences. However, we assumed that each input cell is activated at specific position on a track. In reality, the number of such sparsely activated “landmark” cells is likely to be small, and most of inputs from EC show more complex activity patterns such as grid cells^50^. With such input patterns, place fields cannot be formed by the simple one-to-one association between places and cells. Therefore, we tested whether our model work for grid-like input patterns along the track (see Methods). The spatial frequency and phase of grid patterns were randomly determined for each input neuron to mimic grid cells in EC^50^. An example of input patterns is shown in Fig. 5a. Our two-compartment recurrent network model could learn place code in this environment (Fig. 5b). Furthermore, while the two-compartment model attained high information per spike, the single-compartment model only gained poor information (Fig. 5c). These results suggest that the two-compartment model also works robustly with realistic activity patterns in EC.

### Long-term stability of memory in remote replay events

**Figure 6:**
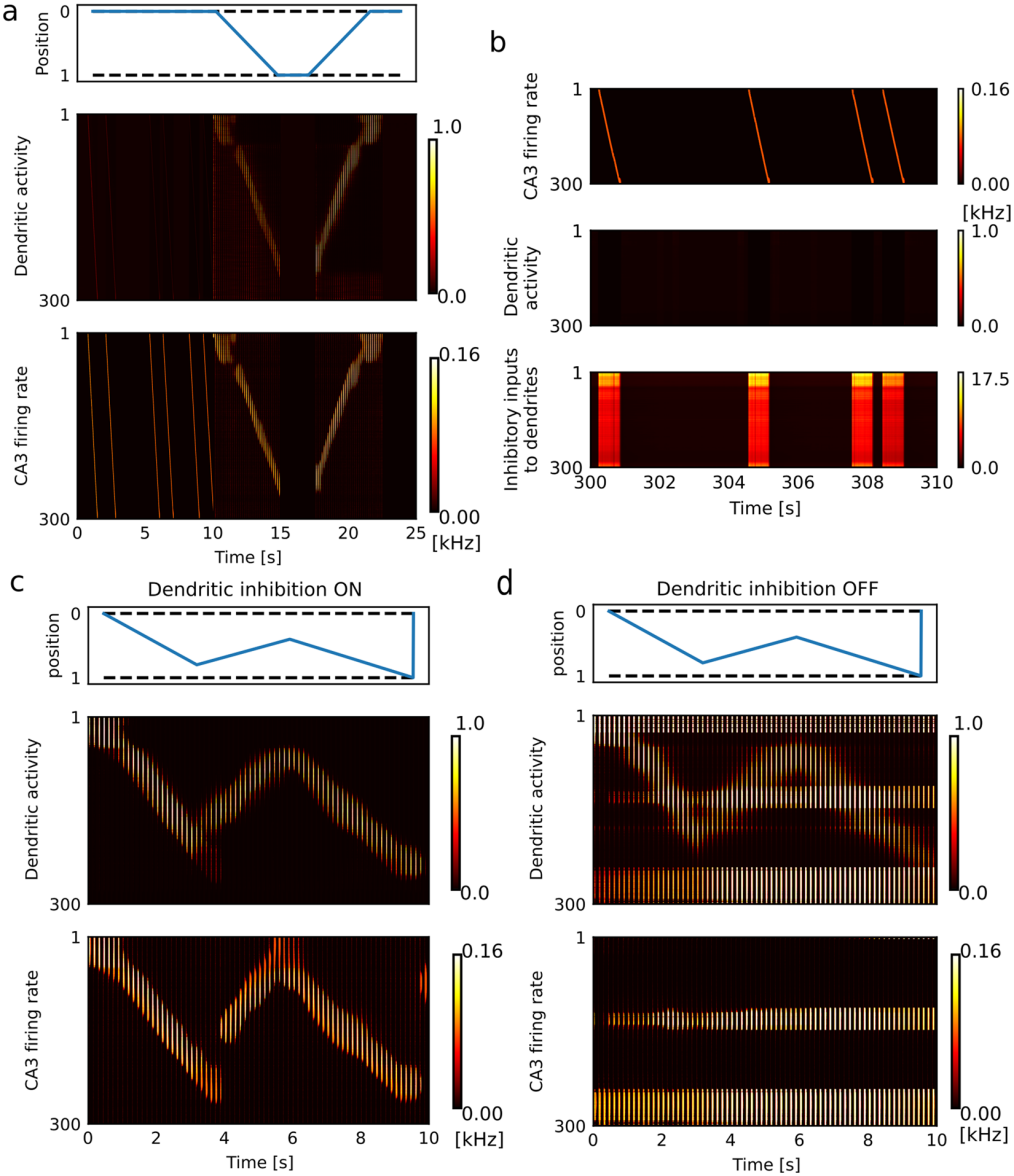
Long-term stability of memory against spontaneous activation. (**a**) Dendritic and somatic activities of the two-compartment CA3 neurons are shown before and during the first traversal on a one-dimensional track. (**b**) Dendritic activity and firing sequences during spontaneous activity are shown together with inhibitory inputs to the dendritic compartments. (**c**) Activity of the two-compartment network model during traversals on the one-dimensional track is shown after exposure to spontaneous activity. (**d**) Similarly, such network activity is shown in the case that the dendritic inhibition was removed during the exposure to spontaneous activity.

At a first glance, memory formation through one-time experience looks easy if synaptic modifications are sufficiently fast. However, this was not the case as there was a trade-off between learning speed and the long-term stability of memory. In the case of spatial memory, previously formed place fields have to be preserved during spontaneous replays in sleep states^6^ and awake replays of remote experiences^7^. Now, we examine the stability of spatial memory against such replay events. To this end, we first trained the network model on an unfamiliar track (Fig. 6a) and then introduced random noise in EC and CA3 to generate irregular firing of EC neurons and spontaneous firing sequences in CA3 (Fig. 6b). We exposed the network to these noisy activities for 600 sec, and then we confirmed that the two-compartment neurons still preserved their place fields (Fig. 6c). This stability was achieved by lateral inhibition between the dendritic compartments, which suppressed dendritic activity during replay events (Figs 3 and 6b, bottom). The inhibitory effect prevented undesirable association of random EC inputs and spontaneous replays in CA3. Actually, the place fields were completely eliminated when all inhibitory weights were set equal to zero during replay events (Fig. 6d). Thus, our two-compartment network model reconciles conflicting demands on the brain’s memory systems, i.e., single-trial formation and long-term stability of memory, without an ad hoc tuning of model parameters.

### Plasticity of dendritic inhibition prevents overwriting of multiple episodes

**Figure 7:**
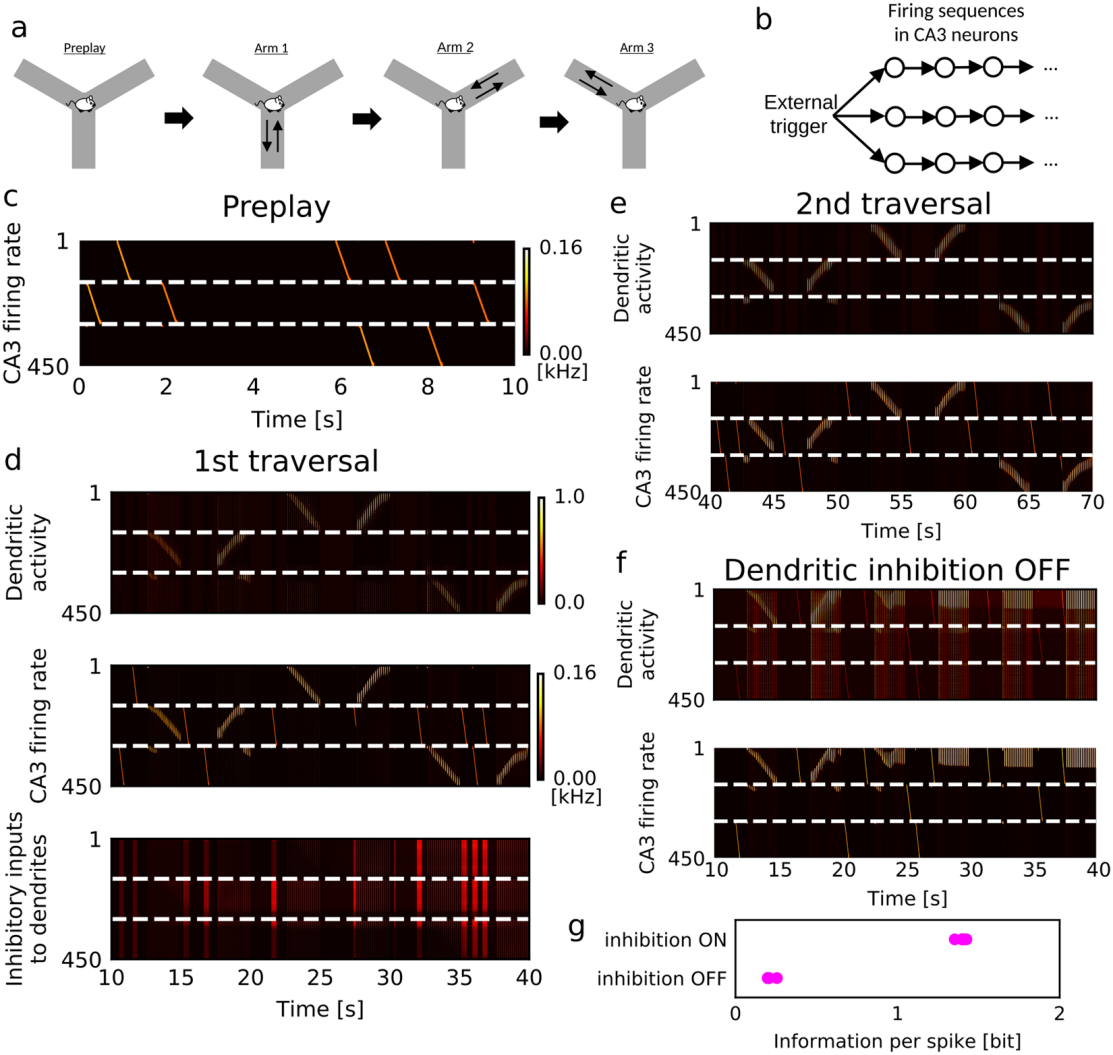
Orthogonal memory formation in a Y-maze. (**a**) Behavioral paradigm is schematically illustrated. The animal starting from the junction successively visits three arms on a Y-maze. (**b**) In the initial setting of the recurrent network, a DG input triggers three firing sequences when the animal is at the junction of the Y-maze. Each branch of sequence consists of 150 neurons. (**c**) Spontaneous activity during the resting state is shown for the three branches of the two-compartment network model before the exploration. (**d**) The dendritic and somatic activities of the two-compartment network model during the first run on the Y-maze. Time evolution of inhibitory inputs to the dendritic compartments is also shown. (**e**) Activity of the two-compartment network model during the second run is shown. (**f**) Activity of two-compartment model without dendritic inhibition during the first run on the Y-maze. (**g**) With and without dendritic inhibition Information per spike was calculated over five simulation trials with different random seeds.

So far, we have studied the one-to-one association between a linear track and a firing sequence. However, in many real-world tasks, the hippocampus has to separately store multiple memories. In spatial navigation experiments, CA3 develops sparse and orthogonal spatial representations^2,3^. To examine whether our two-compartment neuronal network is capable of learning such representations, we tested the formation of spatial memory on a Y-maze, of which three arms were in turn and repeatedly visited by the animal (Fig. 7a). We configured initial recurrent connections such that the CA3 network had three preexisting firing sequences (Fig. 7b), which could be triggered by noise and trigger inputs (Fig. 7c). Here, three is the minimal number of sequences required for learning all three arms. Due to intersomatic lateral inhibition, the trigger inputs could not co-activate all three sequences, and random noise determined which sequence is activated by a trigger input. The firing sequences were accompanied by theta oscillation during run (Fig. 7d–f).

The network model robustly assigned the individual firing sequences to representing different arms (Fig. 7d and e). Inhibitory plasticity played a crucial role in the learning procedure. After the first traversal on an arm, a firing sequence (a CA3 neuron ensemble) was assigned to this arm. When the animal traveled on the second arm, dendritic inhibition decreased the response gain of this neuron ensemble (Fig. 7d, bottom) to associate one of the other neuron ensembles (i.e., other firing sequences) with the second arm. In fact, as shown in Fig. 7f, without dendritic inhibition the different arms may not be represented by different firing sequences. Learning performance assessed by information per spike was significantly degraded without dendritic inhibition (Fig. 7g). By this inhibitory mechanism, this network model encodes a new memory into a yet unassigned firing sequence, avoiding to overwrite old episodes with a novel episode.

### Replay of firing sequences is biased by recent experiences

**Figure 8:**
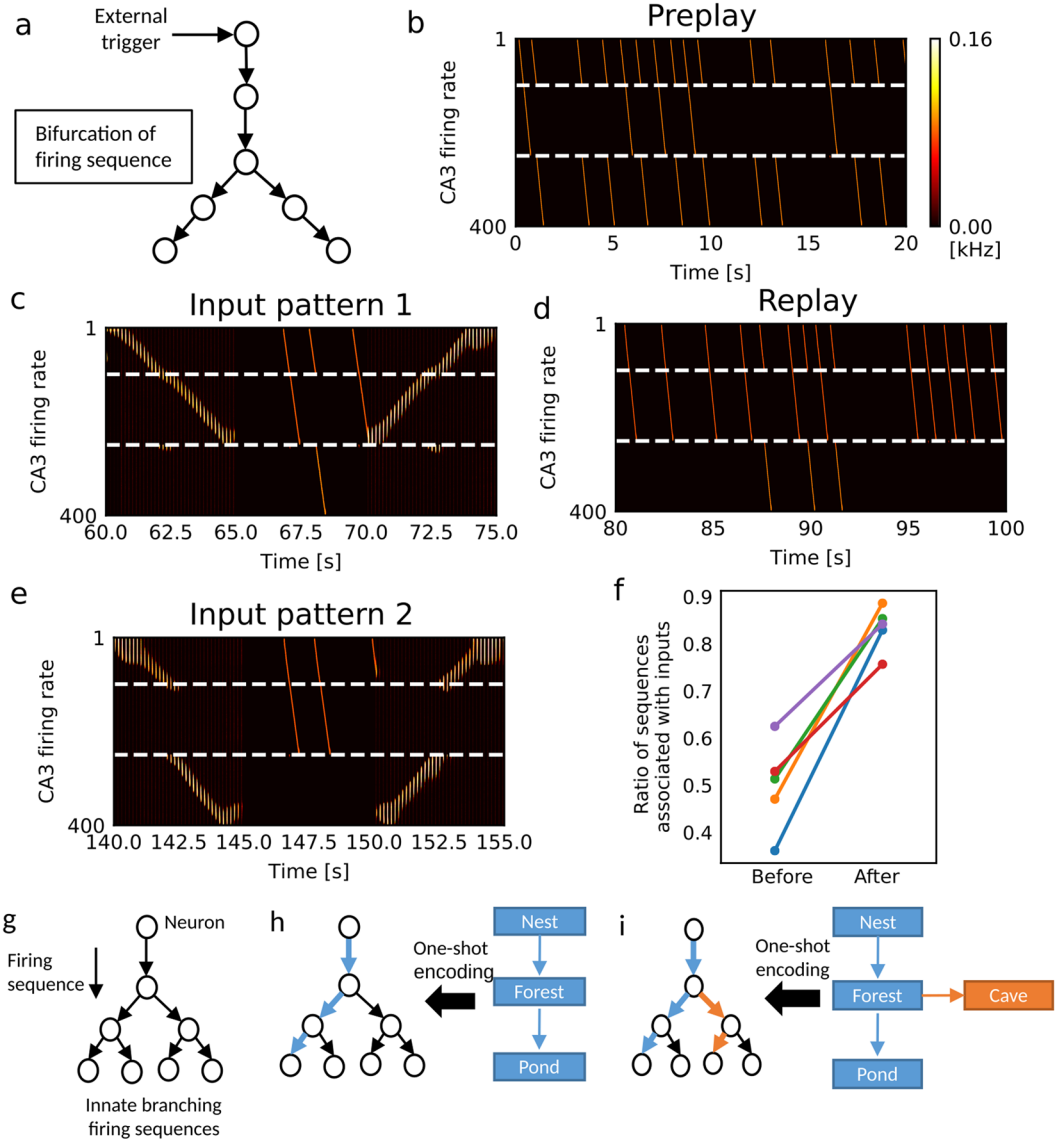
Memory encoding on branching firing sequences. (**a**) The somatic recurrent network of the two-compartment neuron model that has a bifurcating point. Neurons 1 to 100 constitute the trunk, 101 to 250 the left-side branch, and 251 to 400 the right-side branch. (**b**) Spontaneous branching firing sequences before spatial exploration are shown. (**c**) The two-compartment network model associated a sequence of sensory events (input pattern 1) is shown with the left-side branch of synaptic pathways. (**d**) After this encoding, spontaneous replay was biased to the firing sequence associated with input pattern 1. (**e**) The network model encoded a novel sensory sequence (input pattern 2) into the right-side branch of synaptic pathways. (**f**) The relative frequency of replay of the spontaneous firing sequence encoding input pattern 1 was calculated before and after the first experience for five simulation trials using different random seeds. The numbers of sequences propagating into either branch were counted for 60 sec in spontaneous activity. (**g**) The proposed memory encoding model utilizes a rich repertoire of branching firing sequences in the CA3 network. (**h**) Sequential sensory events are associated to a branching of firing sequences. (**i**) Novel sensory events are encoded into a different branch of sequences.

Correlation structure changes in spontaneous hippocampal activity before and after experiences^51^. In particular, replay sequences become statistically significant only after experiences^11^. Does our network model show similar changes? We examined this when the CA3 recurrent network has somewhat complex structure. To be specific, we considered a two-compartment network model with bifurcating firing sequences (Fig. 8a). At the bifurcating point, neurons at the junction were initially connected to both pathways with equal strength, and firing sequences propagated into one of the branches with approximately equal probabilities (Fig. 8b). After the exploration of the 1D track, the model associated one of the branching sequences with this experience (Fig. 8c, input pattern 1) and selectively replayed this sequence in spontaneous activity (Fig. 8d), implying that the recurrent connections for generating this sequence were selectively potentiated. Highly selective replay of the associated sequences was quantified by numerical simulations (Fig. 8f). After the learning, we input a novel sensory sequence (input pattern 2) to the model. Our model encoded input pattern 2 into another branch that was not used for input pattern 1 (Fig. 8e), as in the example in Fig. 7. Notably, to generate input pattern 2, we only shuffled the temporal order of sensory objects (equivalently, the temporal order of firing in EC neurons) of input pattern 1. It implies that our model can discriminate difference in the temporal order of sensory inputs.

The proposed model reconciles the concept of preplay with experience-dependent replay. Assume that CA3 has a rich repertoire of innate firing sequences (Fig. 8g). When the animal experiences sequential sensory events (e.g., from the nest to a pond through a forest), the events are rapidly associated with a branching firing sequence that happens to be most strongly correlated with them (Fig. 8h). This sequence will be replayed more frequently than previously and consolidated more robustly through plasticity at recurrent connections. Now, the animal visited another destination from a midpoint of the learned path (e.g., from the nest to a cave through the forest). Our model suggests that sensory events on the novel path should be assigned to a different branch of firing sequences (Fig. 8i). Then, the merits of this model reside in i) fast and robust memory encoding, ii) economical representations (no need of re-encoding the remembered part, i.e., from the nest to the forest, of novel experiences), and iii) easy update of spatial map (the spatial relationships between old and novel sensory objects are naturally preserved in the branching network structure).

## Discussion

In this study, we showed how spontaneous firing sequences (i.e., preplay events) contribute to cortical memory processing. To show this, we proposed a two-compartment neuron model that incorporates the effects of dendritic spikes on Hebbian learning. The proposed learning rule combines the conventional Hebbian plasticity for PCA of uncorrelated inputs with canonical correlation analysis of correlated somatic and dendritic inputs. A recurrent network of the two-compartment neurons performed robust single-trial learning of sequential sensory events. The model predicts that plasticity of dendritic inhibition plays pivotal roles for the stability and independence of synaptic memory traces. Our results indicate that dendritic computation serves for fast and robust memory encoding.

### Mechanisms and functional implications of CCA-like learning

In the two-compartment neuron, CCA-like learning extracts a minor input component at one compartment when correlated input is given to the other compartment. This computation was inferred from dendritic coincidence detection in neocortical^29,33,34,41^ and hippocampal CA1 pyramidal cells^35,36^, for which back-propagating action potentials play an active role. Though no direct evidence has been reported for coincidence detection in CA3 pyramidal cells, recent studies showed that in these neurons a burst of back-propagating action potentials generates NMDA spikes, which are sufficient for the induction of LTP at CA3-to-CA3 synapses^37^. Therefore, it is not so unrealistic to expect that CA3 pyramidal cells also perform coincidence detection between distal and proximal dendritic inputs.

Similar learning schemes are also expected to work in the neocortex. Actually, the role of spontaneous firing sequences in encoding novel sensory stimuli has been suggested in the auditory and somatosensory cortex^14^. In neocortical pyramidal neurons, calcium spikes presumably integrate the processing of functionally distinct inputs to the basal dendrites and apical tufts^29,34^. Such structure may be useful for optimizing multi-layer feed-forward neural network (deep learning)^52^ and many other types of cortical computation^34^. It has also been shown that dendritic processing is beneficial for sequence processing in the cortex^53^. However, the learning mechanism for linking top-down, bottom-up, and endogenous sequential activity in the cortex is still unknown. We suggest that CCA-like learning is a likely mechanism of this integrated signal processing. In engineering, CCA is a well-established multivariate analysis method used in variety of applications such as the integration of multi-modal sensory inputs in video streams^31^. Our model suggests that cortical neurons can perform similar computations in the brain.

Our CCA-like learning requires dendritic inhibition to ensure the stability and independence of memory traces. Overwriting has been a long-standing issue in memory processing^54^. In our model, activity-dependent inhibitory plasticity at the distal dendrites of pyramidal cells prevents the overwriting of memories. Such plasticity is crucial for the stability of memory traces (Fig. 6) and the robust association of multiple sequential experiences with firing sequences of different neuron ensembles (Figs 7 and 8). Because somatostatin-positive (SOM+) interneurons target the apical dendrites of cortical pyramidal cells^42,43^, this interneuron subtype likely underlies the proposed dendritic inhibition. However, it has been recently shown that each interneuron subtype has a different tendency in connecting with dendritic branches and hence has a different computational effect^55^. It is intriguing to explore how the various subtypes of interneurons differently affect CCA-like learning.

### Realistic network mechanisms for preplay sequences

Local cortical circuits including those of CA3^15,16^ were shown to have log-normal synaptic weight distributions. Computational studies showed that this class of recurrent neuronal networks can generate tremendously many spontaneous firing sequences with various branching patterns^16,19,20^. It will be intriguing to study whether the proposed mechanism can encode complex sensory experiences into realistic spontaneous firing sequences generated by log-normal connectivity. Simulations of such network models will require a spiking version of the two-compartment model as well as an efficient platform for large-scale network simulations. Another possible implementation for generating complex sequential activity is branch-level nonlinear dendritic computing, which increases the robustness and flexibility of recurrent network^53,56,57^. Although learning mechanisms for branch-level computation have been proposed^53,56^, self-organization of spontaneous activity is still challenging.

Realistic recurrent network models will allow us to examine whether the proposed learning scheme generates place fields in a 2D environment. In the previous models^58,59^, Hebbian plasticity reorganized recurrent connections such that omnidirectional 2D place fields emerge from multiple 1D place fields passing through a particular position from various directions. The same mechanism, in principle, works in our model if it has sufficiently many firing sequences for learning various spatial paths. This reorganization of the neural network may recruit new cells for the consolidation of firing sequences, as observed in experiment^12^. However, the formation of stable 2D place fields will be much slower than the learning of 1D tracks.

### Testable assumptions and predictions

The most important assumption of our model is the dendritic mechanism for correlation maximization (CCA), which was modeled based on findings in the neocortex and CA1. Although there are some related experimental studies in CA3^37,60,61^, whether dendritic coincidence detection in CA3 pyramidal cells is analogous to that in CA1 and neocortical pyramidal cells should be clarified by future experiments.

Our model assumes that coincident somatic and dendritic activation potentiates both excitatory and inhibitory synapses. While inhibitory plasticity depends on calcium signals^62,63^, whether it depends on dendritic spikes has yet to be examined. Our results predict that the loss of dendritic inhibition disrupts the stability and orthogonality of CA3 place fields. Whether the removal of dendritic inhibition triggers forgetting or remapping of memory traces before the consolidation is an interesting open question. Selective deletion^64^ or optogenetic inactivation^42^ of SOM+ interneurons may remove dendritic inhibition. Alternatively, activation of vasoactive intestinal polypeptide-positive (VIP) interneurons, which disinhibit distal dendrites^40^, may lead to similar results.

Our model suggests that plasticity of EC-to-CA3 synapses is more important than that of recurrent synapses in CA3 for single-trial learning of place fields. Though the ablation of NMDA receptors in CA3 results in the disruption of pattern completion and single-trial learning^1,65^, which synaptic connections, CA3-to-CA3 synapses or EC-to-CA3 synapses, are more responsible for learning novel experiences has to be clarified. Recently, dopaminergic input from locus coeruleus to CA3 was shown to promote single-trial learning of episodes^66^. Though the underlying mechanisms of the enhanced learning performance remain unknown, one possibility is that the dopaminergic input enhances spontaneous activity, or preplay events, in CA3. It will be intriguing to examine this possibility.

### Relationship to other models of CA3 and dendritic computation

In our model, hippocampal neurons do not have preconfigured place fields, but they are formed through experiences. Previous models learn sequences under the assumption that place fields are configured prior to learning^23–25,27,46^. However, the lack of preconfigured place fields was recently shown in CA1, where artificially induced dendritic spikes generated an arbitrary place field in an arbitrary pyramidal neuron^36^.

Samsonovich and McNaughton^67^ proposed a “map-based path integration” model, which associates sensory inputs with a preexisting hippocampal “chart” (a two-dimensional attractor map). Although this model and ours share a similar concept, our model clarifies the roles of dendritic computation and inhibition in implementing this rapid and robust association. Moreover, the chart model has no plastic recurrent connections and hence does not account for replay events. In Káli and Dayan^59^, recurrent weights were trained through correlations among DG-to-CA3 inputs, and EC-to-CA3 weights through correlations between DG inputs and EC inputs. Thus, their learning rule also produces correlations between EC-to-CA3 inputs and recurrent inputs. However, our learning rule, but not theirs, explains the extremely sparse activity of DG granule cells in spatial exploration^68^ if trigger inputs actually arise from the occasional firing of DG. In addition, our model uses strong recurrent synapses for single-trial learning, while their model requires weak recurrent inputs during the early phase of learning.

Urbanczik and Senn^69^ proposed a two-compartment model in which dendritic synapses are modified to predict somatic activity through unidirectional soma-dendrite interactions. In contrast, our neuron model modifies somatic and dendritic synapses simultaneously through bidirectional soma-dendrite interactions. This raises a conceptual difference between the two models: our model performs unsupervised learning of the two input streams, while their model obeys supervised learning of dendritic input using somatic input as a teacher signal. Recently, dendritic computation and recurrent networks were combined to improve the capacity of pattern completion^70^. In contrast, our model focusses on the role of dendritic computation in sequence learning.

In sum, our multi-compartment learning rule extends the computational ability of neurons to a conjunctive analysis of synaptic inputs targeting different dendritic sites. Because the proximal (somatic) and distal dendrites in pyramidal neurons are targeted by outputs of distinct brain regions, our learning rule has implications for the mechanisms of integrating parallel distributed processes across the brain.

## Methods

### Weight changes and moving thresholds

In all numerical simulations, we modified excitatory synapses in the somatic and dendritic components according to the following second-order stochastic dynamics incorporating delays, weight decays and spontaneous fluctuations:

15 $$\frac{{\rm{d}}}{{\rm{d}}t}{w}_{ij}^{X}={\Delta }{w}_{ij}^{X}-{\eta }_{{\rm{decay}}}{w}_{ij}^{X}+{\sigma }_{w}{\epsilon }(t)\,(X={\rm{som}},{\rm{dnd}}),$$

16 $${\tau }_{{\rm{w}}}\frac{{\rm{d}}}{{\rm{d}}t}{\Delta }{w}_{ij}^{{\rm{som}}}=-{\Delta }{w}_{ij}^{{\rm{som}}}+\eta ((1-\alpha ){x}_{i}(t)({x}_{i}(t)-{\theta }_{i}^{{\rm{som}}})+\alpha {x}_{i}(t){y}_{i}(t))\,(1-{x}_{i}(t))\,{I}_{j}^{{\rm{som}}}(t),$$

17 $${\tau }_{{\rm{w}}}\frac{{\rm{d}}}{{\rm{d}}t}{\Delta }{w}_{ij}^{{\rm{dnd}}}=-\,{\Delta }{w}_{ij}^{{\rm{dnd}}}+\eta ((1-\alpha ){y}_{i}(t)({y}_{i}(t)-{\theta }_{i}^{{\rm{dnd}}})+\alpha {x}_{i}(t){y}_{i}(t))(1-{y}_{i}(t)){I}_{j}^{{\rm{dnd}}}(t),$$

where *τ*_w_ is the time constant for delays of synaptic changes, *η*_decay_ is the speed of weight decay, ${\epsilon }(t)$ is normal Gaussian noise and *σ*_w_ is the standard deviation of spontaneous fluctuation. The weights were constrained in non-negative values during simulations.

Long-term plasticity of dendritic inhibitory weights ${v}_{ij}^{{\rm{dnd}}}$ was implemented as

18 $$\frac{{\rm{d}}}{{\rm{d}}t}{v}_{ij}^{{\rm{dnd}}}={\Delta }{v}_{ij}^{{\rm{dnd}}}-{\eta }_{{\rm{decay}}}{v}_{ij}^{{\rm{dnd}}},$$

19 $${\tau }_{{\rm{w}}}\frac{{\rm{d}}}{{\rm{d}}t}{\Delta }{v}_{ij}^{{\rm{dnd}}}=-\,{\Delta }{v}_{ij}^{{\rm{dnd}}}+{\eta }^{{\rm{inh}}}((1-\alpha ){y}_{i}(t)({y}_{i}(t)-{\theta }^{{\rm{inh}}})+\alpha {x}_{i}(t){y}_{i}(t))(1-{y}_{i}(t)){I}_{j}^{{\rm{dndinh}}}(t).$$

Somatic inhibitory weights ${v}_{i}^{{\rm{som}}}$ were fixed.

For single-compartment neuron, plasticity follows BCM rule:

20 $$\frac{{\rm{d}}}{{\rm{d}}t}{w}_{j}^{X}={\Delta }{w}_{j}^{X}-{\eta }_{{\rm{decay}}}{w}_{i}^{X}+{\sigma }_{w}{\epsilon }(t),$$

21 $${\tau }_{{\rm{w}}}\frac{{\rm{d}}}{{\rm{d}}t}{\Delta }{w}_{j}^{X}=-{\Delta }{w}_{j}^{X}+\eta x(t)(x(t)-{\theta }^{{\rm{som}}})(1-x(t)){I}_{j}^{X}(t).$$

Moving thresholds for BCM theory were defined as ${\theta }_{i}^{{\rm{som}}}={c}_{0}{({E}_{i}^{{\rm{som}}})}^{2},{\theta }_{i}^{{\rm{dnd}}}={c}_{0}{({E}_{i}^{{\rm{dnd}}})}^{2}$. We updated the mean activities ${E}_{i}^{{\rm{s}}{\rm{o}}{\rm{m}}},\,{E}_{i}^{{\rm{d}}{\rm{n}}{\rm{d}}}$ by solving

22 $${\tau }_{{\rm{mean}}}\frac{{\rm{d}}}{{\rm{d}}t}{E}_{i}^{{\rm{som}}}=-\,{E}_{i}^{{\rm{som}}}+x(t),$$

23 $${\tau }_{{\rm{mean}}}\frac{{\rm{d}}}{{\rm{d}}t}{E}_{i}^{{\rm{dnd}}}=-\,{E}_{i}^{{\rm{dnd}}}+y(t),$$

where *τ*_mean_ determines the typical time scale of the averaging.

Parameters were ${c}_{0}=70,{\tau }_{{\rm{w}}}=1000\,{\rm{ms}},{\sigma }_{{\rm{w}}}=0.001,\,{\eta }_{{\rm{decay}}}={10}^{-7}$ and ${\tau }_{{\rm{mean}}}=60000\,{\rm{ms}}$ unless otherwise specified.

### Inhibitory feedback model

We implemented inhibitory feedback to pyramidal neuron *i* as $\sum _{j}{v}_{ij}^{{\rm{som}}}{I}_{j}^{{\rm{sominh}}}(t)$ (somatic inhibition) and $\sum _{j}{v}_{ij}^{{\rm{dnd}}}{I}_{j}^{{\rm{dndinh}}}(t)$ (dendritic inhibition). Somatic inhibitory weights ${v}_{ij}^{{\rm{som}}}$ were fixed throughout the present simulations, and dendritic inhibitory weights ${v}_{ij}^{{\rm{dnd}}}$ were modified by the plasticity rule given in Eqs (18) and (19). Outputs of inhibitory neurons ${I}_{i}^{{\rm{sominh}}}(t)$ and ${I}_{i}^{{\rm{dndinh}}}(t)$ were determined by the summed outputs of excitatory neurons in the recurrent network as

24 $${I}^{{\rm{sominh}}}(t)=\sum _{j}{\theta }_{ij}^{{\rm{som}}}{I}_{j}^{{\rm{PY}}}(t),$$

25 $${I}^{{\rm{dndinh}}}(t)=\sum _{j}{\theta }_{ij}^{{\rm{dnd}}}{I}_{j}^{{\rm{PY}}}(t).$$

where ${I}_{j}^{{\rm{PY}}}(t)$ are unweighted synaptic outputs from pyramidal neurons that are calculated by the same way with ${I}_{j}^{{\rm{som}}}(t)$ in Eq. (4) or Eq. (29) using ${u}_{j}^{{\rm{som}}}(t)={z}_{j}(t)$. The weights ${\theta }_{ij}^{{\rm{som}}}$ and ${\theta }_{ij}^{{\rm{dnd}}}$ were uniformly sampled from [0, 1] and normalized to satisfy $\sum _{i}{\theta }_{ij}^{{\rm{s}}{\rm{o}}{\rm{m}}}=\sum _{i}{\theta }_{ij}^{{\rm{d}}{\rm{n}}{\rm{d}}}={{N}_{inh}}^{-1}$. These weights were fixed during all simulations. The number of inhibitory inputs was 1 in Fig. 3 and 100 in other simulations for either of somatic inhibition and dendritic inhibition.

### Two-compartment recurrent neural network model

Here we define the two-compartment neural network used in Figs 4 to 8. The activity of neuron *i* in two-compartment recurrent neural networks was described as

26 $${x}_{i}(t)={\rm{f}}(\sum _{j}{w}_{ij}^{{\rm{som}}}{I}_{j}^{{\rm{som}}}(t)-\sum _{j}{v}_{ij}^{{\rm{som}}}{I}_{j}^{{\rm{sominh}}}(t)+\beta {y}_{i}(t-{\rm{\Delta }}t)+{I}_{i}^{{\rm{ext}}}(t)),$$

27 $${y}_{i}(t)={\rm{f}}(\sum _{j}{w}_{ij}^{{\rm{dnd}}}{I}_{j}^{{\rm{dnd}}}(t)-\sum _{j}{v}_{ij}^{{\rm{dnd}}}{I}_{j}^{{\rm{dndinh}}}(t)+\beta {x}_{i}(t-{\rm{\Delta }}t)),$$

28 $${z}_{i}(t)=(1+\gamma {y}_{i}(t))\varphi {x}_{i}(t),$$

where parameters were set as $\varphi =0.08\,{\rm{kHz}},{\theta }_{f}=5,\,\beta =2.5,\,{\rm{and}}\,\gamma =1$ unless otherwise specified. ${I}_{i}^{{\rm{ext}}}(t)$ is external input, which varied depending on the simulation settings. Synaptic inputs ${I}_{j}^{X}(t)$ were calculated from recurrent inputs ${u}_{j}^{{\rm{som}}}(t)={z}_{j}(t)$ and firing rates of EC neurons ${u}_{j}^{{\rm{dnd}}}(t)$ as

29 $$\frac{{\rm{d}}}{{\rm{d}}t}{I}_{j}^{X}(t)=-\frac{1}{{\tau }_{{\rm{L}}}}{I}_{j}^{X}(t)+{u}_{j}^{X}(t){D}_{j}^{X}(t){F}_{j}^{X}(t)\,(X={\rm{som}},{\rm{dnd}}),$$

where ${D}_{j}^{X}(t)$ and ${F}_{j}^{X}(t)$ are variables for short-term synaptic plasticity:

30 $$\frac{{\rm{d}}}{{\rm{d}}t}{D}_{j}^{X}(t)=\frac{1-{D}_{j}^{X}(t)\,}{{\tau }_{{\rm{STD}}}}-{u}_{j}^{X}(t){D}_{j}^{X}(t){F}_{j}^{X}(t),$$

31 $$\frac{{\rm{d}}}{{\rm{d}}t}{F}_{j}^{X}(t)=\frac{{U}_{{\rm{STF}}}-{F}_{j}^{X}(t)}{{\tau }_{{\rm{STF}}}}+{U}_{{\rm{STF}}}(1-{F}_{j}^{X}(t)){u}_{j}^{X}(t).$$

The values of parameters were set as ${\tau }_{{\rm{STD}}}=500\,{\rm{ms}},{\tau }_{{\rm{STF}}}=200\,{\rm{ms}}$ and ${\tau }_{{\rm{L}}}=10\,{\rm{ms}}$. The value of initial release probability *U*_STF_ was 0.5 for all excitatory synapses in the immobile state of animal, and was changed to 0.03 for recurrent synapses during animal’s movement. At the moment that the animal started a movement from immobile state, ${F}_{j}^{{\rm{som}}}(t)$ was immediately changed to 0.03. We note that the long-term plasticity rules also depend on short-term plasticity through ${I}_{j}^{X}(t)$.

The firing rate of EC neurons ${u}_{j}^{{\rm{dnd}}}(t)$ were calculated as

32 $${u}_{j}^{{\rm{dnd}}}(t)={\varphi }_{{\rm{input}}}f({I}_{j}^{{\rm{input}}}(t)),$$

where ${\varphi }_{{\rm{input}}}=0.08\,{\rm{kHz}}$. ${I}_{j}^{{\rm{input}}}(t)$ depends on simulation settings.

The values of parameters for plasticity were *α* = 0.9 and *η* = *η*^inh^ = 1. Self-connections ${w}_{ii}^{som}$ were fixed at zero. Simulation without dendritic inhibition was performed with *η*^inh^ = 0.

### Single-compartment recurrent neural network model

In single-compartment recurrent neural networks, all somatic and dendritic inputs were connected to a single compartment (soma). Accordingly, the activity of neuron *i* was described as

33 $${x}_{i}(t)={\rm{f}}(\sum _{j}{w}_{ij}^{{\rm{som}}}{I}_{j}^{{\rm{som}}}(t)+\sum _{j}{w}_{ij}^{{\rm{dnd}}}{I}_{j}^{{\rm{dnd}}}(t)-\sum _{j}{v}_{ij}^{{\rm{som}}}{I}_{j}^{{\rm{sominh}}}(t)+{I}_{i}^{{\rm{ext}}}(t)),$$

34 $${z}_{i}(t)=\varphi {x}_{i}(t).$$

Variables in this model were calculated in the same way to those in the two-compartment model. We updated both somatic and dendritic excitatory weights ${w}_{ij}^{{\rm{som}}}$ and ${w}_{ij}^{{\rm{dnd}}}$ by BCM theory with somatic activity. The values of parameters for the single-compartment model was basically the same as those of the two-compartment model, except $\varphi =0.1\,{\rm{kHz}}$. Learning speed was set as *η* = 0.5 in Fig. 5d, though different values (*η* = 0.1, 1.0) were also used in a quantitative assessment.

### Details of the single-cell simulations

In Figs 1 and 2, four independent source signals *s*_*i*_(*t*)(*i* = 1, 2, 3, 4) were generated from Ornstein-Uhlenbeck process

35 $$\frac{{\rm{d}}}{{\rm{d}}t}{s}_{i}(t)=-\,\frac{1}{{\tau }_{{\rm{s}}}}{s}_{i}(t)+{\sigma }_{{\rm{s}}}{\epsilon }(t),$$

where ${\tau }_{{\rm{s}}}=10\,{\rm{ms}},{\sigma }_{{\rm{s}}}=0.1$ and ${\epsilon }(t)$ is normal Gaussian noise. Input currents to somatic input neurons ${I}_{j}^{{\rm{input}},{\rm{som}}}(t)$ were determined as

36 $$\frac{{\rm{d}}}{{\rm{d}}t}{I}_{j}^{{\rm{input}},{\rm{som}}}(t)=-\,\frac{1}{{\tau }_{{\rm{L}}}}{I}_{j}^{{\rm{input}},{\rm{som}}}(t)+{s}_{1}(t)+{\sigma }_{{\rm{n}}}{\epsilon }(t)\,(j\in {\rm{groupA}}),$$

37 $$\frac{{\rm{d}}}{{\rm{d}}t}{I}_{j}^{{\rm{input}},{\rm{som}}}(t)=-\,\frac{1}{{\tau }_{{\rm{L}}}}{I}_{j}^{{\rm{input}},{\rm{som}}}(t)+{s}_{3}(t)+{\sigma }_{{\rm{n}}}{\epsilon }(t)\,(j\in {\rm{groupB}}),$$

where *σ*_n_ = 0.1. Input currents to dendritic input neurons ${I}_{j}^{{\rm{input}},{\rm{dnd}}}(t)$ were was determined as

38 $$\frac{{\rm{d}}}{{\rm{d}}t}{I}_{j}^{{\rm{input}},{\rm{dnd}}}(t)=-\,\frac{1}{{\tau }_{{\rm{L}}}}{I}_{j}^{{\rm{input}},{\rm{dnd}}}(t)+{s}_{{\rm{A}}^{\prime} }(t)+{\sigma }_{{\rm{n}}}{\epsilon }(t)\,(j\in {\rm{groupA}}^{\prime} ),$$

39 $$\frac{{\rm{d}}}{{\rm{d}}t}{I}_{j}^{{\rm{input}},{\rm{dnd}}}(t)=-\,\frac{1}{{\tau }_{{\rm{L}}}}{I}_{j}^{{\rm{input}},{\rm{dnd}}}(t)+{s}_{4}(t)+{\sigma }_{{\rm{n}}}{\epsilon }(t)\,(j\in {\rm{groupB}}^{\prime} ).$$

In the case of uncorrelated A and A’, ${s}_{{\rm{A}}^{\prime} }(t)={s}_{1}(t)$ while in the case of correlated A and A’, ${s}_{{\rm{A}}^{\prime} }(t)={s}_{2}(t)$. Output firing rates of input neurons ${u}_{j}^{{\rm{som}}}(t),$ ${u}_{j}^{{\rm{dnd}}}(t)$ were calculated by the same sigmoidal function f(*I*) as that of the two-compartment neuron model:

40 $${u}_{j}^{{\rm{som}}}(t)={\varphi }_{{\rm{input}}}{\rm{f}}({I}_{j}^{{\rm{input}},{\rm{som}}}(t)),$$

41 $${u}_{j}^{{\rm{dnd}}}(t)={\varphi }_{{\rm{input}}}{\rm{f}}({I}_{j}^{{\rm{input}},{\rm{dnd}}}(t)).$$

The values of parameters were given as $\varphi ={\varphi }_{{\rm{input}}}=0.08\,{\rm{kHz}},\alpha =0.5,\beta =0,\gamma =1,\eta =0.2\,$and *σ*_w_ = 0.005. Simulations of the single-compartment neuron were performed for *α* = *β* = *γ* = 0 without changing the values of the other parameters. Initial weights were uniformly sampled from [0, 5].

### Details of simulations of inhibitory feedback model

In Fig. 3, we calculated source signals *s*_*i*_(*t*) in the same way with previous section. In the two-cell simulation for separation, we prepared two source signals. We calculated ${I}_{j}^{{\rm{input}},{\rm{som}}}(t)\,(1\le j\le 10)$ and ${I}_{j}^{{\rm{input}},{\rm{dnd}}}(t)\,(1\le j\le 20)$ by

42 $$\frac{{\rm{d}}}{{\rm{d}}t}{I}_{j}^{{\rm{input}},{\rm{som}}}(t)=-\,\frac{1}{{\tau }_{{\rm{L}}}}{I}_{j}^{{\rm{input}},{\rm{som}}}(t)+{s}_{1}(t)+{s}_{2}(t)+{\sigma }_{{\rm{n}}}{\epsilon }(t).$$

43 $$\frac{{\rm{d}}}{{\rm{d}}t}{I}_{j}^{{\rm{input}},{\rm{dnd}}}(t)=-\,\frac{1}{{\tau }_{{\rm{L}}}}{I}_{j}^{{\rm{input}},{\rm{dnd}}}(t)+{s}_{1}(t)+{\sigma }_{{\rm{n}}}{\epsilon }(t)\,(j\in {\rm{groupA}}),$$

44 $$\frac{{\rm{d}}}{{\rm{d}}t}{I}_{j}^{{\rm{input}},{\rm{dnd}}}(t)=-\,\frac{1}{{\tau }_{{\rm{L}}}}{I}_{j}^{{\rm{input}},{\rm{dnd}}}(t)+{s}_{2}(t)+{\sigma }_{{\rm{n}}}{\epsilon }(t)\,(j\in {\rm{groupB}}).$$

In the single-cell simulation for stabilization, we prepared 21 source signals. Throughout the simulation, we calculated ${I}_{j}^{{\rm{input}},{\rm{som}}}(t)\,(1\le j\le 10)$ and ${I}_{j}^{{\rm{input}},{\rm{dnd}}}(t)\,(1\le j\le 20)$ by

45 $$\frac{{\rm{d}}}{{\rm{d}}t}{I}_{j}^{{\rm{input}},{\rm{som}}}(t)=-\,\frac{1}{{\tau }_{{\rm{L}}}}{I}_{j}^{{\rm{input}},{\rm{som}}}(t)+{s}_{1}(t)+{\sigma }_{{\rm{n}}}\epsilon (t),$$

46 $$\frac{{\rm{d}}}{{\rm{d}}t}{I}_{j}^{{\rm{input}},{\rm{dnd}}}(t)=-\,\frac{1}{{\tau }_{{\rm{L}}}}{I}_{j}^{{\rm{input}},{\rm{dnd}}}(t)+{s}_{{\rm{j}}+1}(t)+{\sigma }_{{\rm{n}}}{\epsilon }(t),$$

from 0 s to 300 s,

47 $$\frac{{\rm{d}}}{{\rm{d}}t}{I}_{j}^{{\rm{input}},{\rm{dnd}}}(t)=-\,\frac{1}{{\tau }_{{\rm{L}}}}{I}_{j}^{{\rm{input}},{\rm{dnd}}}(t)+{s}_{1}(t)+{\sigma }_{{\rm{n}}}{\epsilon }(t)\,(j\in {\rm{groupA}}),$$

48 $$\frac{{\rm{d}}}{{\rm{d}}t}{I}_{j}^{{\rm{input}},{\rm{dnd}}}(t)=-\,\frac{1}{{\tau }_{{\rm{L}}}}{I}_{i}^{{\rm{input}},{\rm{dnd}}}(t)+{s}_{{\rm{j}}+1}(t)+{\sigma }_{{\rm{n}}}{\epsilon }(t)\,(j\in {\rm{groupB}}),$$

from 300 s to 600 s, and

49 $$\frac{{\rm{d}}}{{\rm{d}}t}{I}_{j}^{{\rm{input}},{\rm{dnd}}}(t)=-\,\frac{1}{{\tau }_{{\rm{L}}}}{I}_{j}^{{\rm{input}},{\rm{dnd}}}(t)+{s}_{{\rm{j}}+1}(t)+{\sigma }_{{\rm{n}}}{\epsilon }(t)\,(j\in {\rm{groupA}}),$$

50 $$\frac{{\rm{d}}}{{\rm{d}}t}{I}_{j}^{{\rm{input}},{\rm{dnd}}}(t)=-\,\frac{1}{{\tau }_{{\rm{L}}}}{I}_{j}^{{\rm{input}},{\rm{dnd}}}(t)+{s}_{1}(t)+{\sigma }_{{\rm{n}}}{\epsilon }(t)\,(j\in {\rm{groupB}}),$$

from 600 s to 1200 s. We determined output firing rates of input neurons ${u}_{j}^{{\rm{som}}}(t),$ ${u}_{j}^{{\rm{dnd}}}(t)$ by Eq. (40), (41).

The values of parameters for the two-compartment neuron model were given as $\varphi ={\varphi }_{{\rm{input}}}=0.08\,{\rm{kHz}},\alpha =0.9,$ $\beta =2.5,\gamma =1$ and *η* = *η*^inh^ = 0.2. Initial excitatory weights were uniformly sampled from [0, 5] and dendritic inhibitory weights ${v}_{i}^{{\rm{dnd}}}(t)$ were initially zero. Somatic inhibitory weights ${v}_{i}^{{\rm{som}}}(t)$ were fixed at 20. Simulations without dendritic inhibition were performed with *η*^inh^ = 0.

### Simulation settings for the one-dimensional track

In Fig. 4, we used 300 CA3 neurons and 500 EC neurons. Initial recurrent synaptic weights from neuron *j* to neuron *i* (*i* ≠ *j*) in CA3 were given as

51 $${w}_{ij}^{{\rm{som}}}={w}_{{\rm{\max }}}\exp (-0.5{(\frac{i-j}{{w}_{{\rm{width}}}})}^{2}),$$

where *w*_max_ = 18, *w*_width_ = 5. Here we included random fluctuation of weights sampled from normal Gaussian distribution $\,{\epsilon }(t)$, and negative weights were set to zero. Self-connections ${w}_{ii}^{som}$ were always zero. In qualitative assessment, we multiplied 0.5 or 1.25 to *w*_max_ in each simulation.

Initial synaptic weights from EC ${w}_{ij}^{{\rm{dnd}}}$ were determined as

52 $${w}_{ij}^{{\rm{dnd}}}={w}_{{\rm{\max }}}^{{\rm{dnd}}}\exp (-0.5{(\frac{i-j}{{w}_{{\rm{width}}}})}^{2}).$$

where ${w}_{{\rm{\max }}}^{{\rm{dnd}}}=5$. We used this setting to simulate the “familiar track”. In the simulation of “unfamiliar track”, we randomly shuffled values of these weights in each postsynaptic neuron *i*. Namely, shuffling was performed for index *j*.

A function satisfying $0\le pos(t)\le 1$ designated the animal’s position on the one-dimensional track. The animal stopped at *pos*(*t*) = 0 from 0 s to 5 s. From 10 s to 25 s, the position in first run is expressed as

53 $$pos(t)=\{\begin{array}{l}\frac{t-15}{5}(10s\le t < 15s)\\ 1(15s\le t < 17.5s)\\ 1-\frac{t-17.5}{5}(17.5s\le t < 22.5s)\\ 0(22.5s\le t < 25s)\end{array}.$$

From 25 s to 40 s, the position in second run is expressed as

54 $$pos(t)=\{\begin{array}{l}\frac{t-25}{10}(25s\le t < 35s)\\ 1-\frac{t-35}{2.5}(35s\le t < 37.5s).\\ 0(37.5s\le t < 40s)\end{array}$$

From 40 s to 50 s, the position in third run is expressed as

55 $$pos(t)=\{\begin{array}{l}0.8\times \frac{t-40}{4}(40s\le t < 44s)\\ 0.8-0.4\times \frac{t-44}{3}(44s\le t < 47s)\\ 0.4+0.6\times \frac{t-47}{3}(47s\le t < 50s)\end{array}.$$

External inputs to somatic compartments ${I}_{i}^{{\rm{ext}}}(t)$ were

56 $${I}_{i}^{{\rm{ext}}}(t)=\{\begin{array}{cc}{I}^{{\rm{theta}}}(t)+{I}^{{\rm{trig}}}(t)+{n}_{i}^{{\rm{ext}}}(t), & {\rm{if}}\,1\le i\le 10\\ {I}^{{\rm{theta}}}(t)-{I}^{{\rm{trig}}}(t)+{n}_{i}^{{\rm{ext}}}(t), & {\rm{otherwise}}\end{array}.$$

*I* ^trig^(*t*) is a trigger input for firing sequences, which takes 10 or 0. When the animal was immobile, the trigger was activated by a Poisson process at 1 Hz, and the duration of each activation was 10 ms. Additionally, when the animal started the first run in each simulation trial, we turned on the trigger for 100 ms. Note that we did not induce any trigger when the animal started the second and later runs. *I* ^theta^(*t*) stands for theta oscillatory input from medial septum

57 $${I}^{{\rm{theta}}}(t)={A}_{{\rm{theta}}}\,\sin (2\pi {f}_{{\rm{theta}}}t),$$

during run and *I* ^theta^(*t*) = 0 during immobility. Values of parameters were set as *A*_theta_ = 10 and *f*_theta_ = 7/1000 kHz. Noise term ${n}_{i}^{{\rm{ext}}}(t)$ were generated by

58 $$\frac{{\rm{d}}}{{\rm{d}}t}{n}_{i}^{{\rm{ext}}}(t)=-\,\frac{1}{{\tau }_{{\rm{L}}}}{n}_{i}^{{\rm{ext}}}(t)+{\sigma }_{{\rm{n}}}{\epsilon }(t),$$

where *σ*_n_ = 0.1 and ${\epsilon }(t)$ is Gaussian white noise.

Inputs to EC neurons ${I}_{i}^{{\rm{input}}}(t)\,(1\le i\le 500)$ were given as

59 $${I}_{i}^{{\rm{input}}}(t)={n}_{i}^{{\rm{input}}}(t),$$

during immobility, where noise term ${n}_{i}^{{\rm{input}}}(t)$ followed the same dynamics as ${n}_{i}^{{\rm{ext}}}(t)$. During run, inputs to position-dependent EC neurons ${I}_{i}^{{\rm{input}}}(t)\,(1\le i\le 300)$ were given as

60 $${I}_{i}^{{\rm{input}}}(t)={A}_{{\rm{F}}}\exp (-0.5{(\frac{pos(t)-center(i)}{{\sigma }_{{\rm{F}}}})}^{2})+0.5{I}^{{\rm{theta}}}(t)-0.5+{n}_{i}^{{\rm{input}}}(t),$$

where ${\epsilon }(t)$ is Gaussian white noise and *center*(*i*) = *i*/300. Inputs to distractor EC neurons (301 ≤ *i* ≤ 500) during run were given as

61 $${I}_{i}^{{\rm{input}}}(t)={s}^{{\rm{dist}}}(t)+0.5{I}^{{\rm{theta}}}(t)-\,0.5+{n}_{i}^{{\rm{input}}}(t),$$

Sources for distractors *s*^dist^(*t*) were generated from independent Ornstein-Uhlenbeck processes

62 $$\frac{{\rm{d}}}{{\rm{d}}t}{s}^{{\rm{dist}}}(t)=-\,\frac{1}{{\tau }_{{\rm{dist}}}}{s}^{{\rm{dist}}}(t)+{\sigma }_{{\rm{s}}}^{{\rm{dist}}}{\epsilon }(t).$$

Values of parameters were set as *σ*_n_ = 1, *A*_F_ = 5.0, *σ*_F_ = 0.1, *σ*_dist_ = 0.02 and τ_dist_ = 500 ms.

When gain modulation was turned off (*β* = 4, *γ* = 0), the maximum firing rate *ϕ* was changed from 0.08 kHz to 0.1 kHz.

### Learning place fields from grid-cell activity

In Fig. 5, simulation setting was basically the same as in Fig. 4 but the number of input neurons was changed to 300. Among these neurons, 100 neurons were distractors and 200 neurons were grid cells, which were activated as

63 $${I}_{i}^{{\rm{i}}{\rm{n}}{\rm{p}}{\rm{u}}{\rm{t}}}(t)={A}_{{\rm{g}}{\rm{r}}{\rm{i}}{\rm{d}}}\exp ({c}_{{\rm{g}}{\rm{r}}{\rm{i}}{\rm{d}}}\,\cos (\frac{2\pi pos(t)}{{w}_{i}^{{\rm{g}}{\rm{r}}{\rm{i}}{\rm{d}}}}-{p}_{i}^{{\rm{g}}{\rm{r}}{\rm{i}}{\rm{d}}}))+0.5{I}^{{\rm{t}}{\rm{h}}{\rm{e}}{\rm{t}}{\rm{a}}}(t)-0.5+{n}_{i}^{{\rm{i}}{\rm{n}}{\rm{p}}{\rm{u}}{\rm{t}}}(t),$$

during run. The same parameter values *A*_grid_ = 5 and *c*_grid_ = 3 were used for all grid cells, whereas ${p}_{i}^{{\rm{g}}{\rm{r}}{\rm{i}}{\rm{d}}}$ and ${w}_{i}^{{\rm{g}}{\rm{r}}{\rm{i}}{\rm{d}}}$ were sampled independently and randomly for different grid cells from [0, 2*π*] and [0.2, 0.6], respectively.

### Simulation settings for spontaneous replay

In Fig. 6, initial setting was the same as in Fig. 4 but there was no distractor inputs. Only the first run was performed on the one-dimensional track, and spontaneous activity was simulated for the next 600 s. After that, simulation of the “third run” in Fig. 4 was conducted. During spontaneous activity, simulation setting was basically the same as that of immobility periods in Fig. 4 except for the addition of population bursts in EC, which were simulated by adding inputs ${n}_{i}^{{\rm{burst}}}(t)$ as

64 $${I}_{i}^{{\rm{input}}}(t)={s}^{{\rm{burst}}}(t){n}_{i}^{{\rm{burst}}}(t)+{n}_{i}^{{\rm{input}}}(t),$$

65 $$\frac{{\rm{d}}}{{\rm{d}}t}{n}_{i}^{{\rm{burst}}}(t)=-\,\frac{1}{{\tau }_{{\rm{burst}}}}{n}_{i}^{{\rm{burst}}}(t)+{\sigma }_{{\rm{burst}}}{\epsilon }(t).$$

where *σ*_burst_ = 0.1 and *τ*_burst_ = 100 ms. We turned on and off the burst input by switching *s*^burst^(*t*) between 1 and 0. The occurrence of population bursts followed a Poisson process at 1 Hz, and each burst lasted for 200 ms. For the results shown in Fig. 6d (dendritic inhibition OFF), we performed the same procedure but all weights of the dendritic inhibition and *η*^inh^ was set to zero after the “first run”.

### Simulation settings for the Y-shape track

In Fig. 7, we used 450 CA3 neurons. We divided these neurons into three groups, $1\le i\le 150,151\le i\le 300,301\le i\le 450$, and recurrent synaptic weights within each group were determined in the same way as in the one-dimensional track, using *w*_max_ = 20 and *w*_width_ = 5. Recurrent synaptic weights across groups were initially zero. Initial synaptic weights from EC ${w}_{ij}^{{\rm{dnd}}}$ were uniformly sampled from the interval [0, 2].

The current position of the animal on the Y-shape track was specified by the arm number (*arm*(*t*) = 1, 2, 3) and the position on the current arm, $0\le pos(t)\le 0.5$. During the first 10 s, the animal stopped at the center of the Y-shape arm. After that, the animal repeated the following movement on different arms every 10 s:

66 $$pos(t)=\{\begin{array}{l}0\,(0s\le t < 2.5s)\\ 0.5\times \frac{t-2.5}{2.5}\,(2.5s\le t < 5s)\\ 0.5\,(5s\le t < 7.5s)\\ 0.5-0.5\times \frac{t-7.5}{2.5}\,(7.5s\le t < 10s)\end{array}.$$

External inputs to the somatic compartments ${I}_{i}^{{\rm{ext}}}(t)$ were basically the same as the ones used for the one-dimensional track. However, trigger inputs (*I* ^trig^(*t*) = 5 or 0) were positively induced to activate 10 neurons per group ($1\le i\le 10,151\le i\le 160,301\le i\le 310$) for 200 ms when the animal started to run each arm for the first time in the simulation.

We used 450 position-dependent EC neurons, and inputs to these EC neurons ${I}_{i}^{{\rm{input}}}(t)$ (1 ≤ *i* ≤ 450) during immobility were the same as those in the one-dimensional track, whereas the inputs during the run depended on animal’s position as

67 $${I}_{i}^{{\rm{input}}}(t)={I}_{i}^{{\rm{pos}}}(t)+0.5{I}^{{\rm{theta}}}(t)-0.5+{n}_{i}^{{\rm{input}}}(t),$$

68 $${I}_{i}^{{\rm{pos}}}(t)=\{\begin{array}{ll}{A}_{{\rm{F}}}\exp (-0.5{(\frac{pos(t)-center(i)}{{\sigma }_{{\rm{F}}}})}^{2}), & if\,arm(t)=center\_arm(i)\\ 0, & otherwise\end{array},$$

where the receptive field center *center*(*i*) of neuron *i* was uniformly sampled from [0, 0.5] and 150 neurons were assigned to each arm: *center*_*arm*(*i*) =  1, 2 and 3 for 1 ≤ *i* ≤ 150, 151 ≤ *i* ≤ 300, and 301 ≤ *i* ≤ 450, respectively.

### Simulation settings for branching firing sequences

In Fig. 8, we used 400 CA3 neurons and 300 EC neurons. We divided neurons into three groups, $1\le i\le 100\,({\rm{root}}),101\le i\le 250\,({\rm{branch}}\,{\rm{A}}),251\le i\le 400\,({\rm{branch}}\,{\rm{B}})$, and recurrent synaptic weights within each group were determined in the same way with the one-dimensional track, using *w*_max_ = 18 and *w*_width_ = 5. Weights were set to zero between branch A and branch B, and

69 $${w}_{ij}^{{\rm{som}}}={w}_{ji}^{{\rm{som}}}={w^{\prime} }_{\max }\exp (-0.5{(\frac{i-j}{{w}_{{\rm{width}}}})}^{2})\,(i\in {\rm{root}},\,j\in {\rm{branch}}\,{\rm{A}}),$$

70 $${w}_{ij}^{{\rm{som}}}={w}_{ji}^{{\rm{som}}}={w^{\prime} }_{\max }\exp (-0.5{(\frac{(100-i)+(j-250)}{{w}_{{\rm{width}}}})}^{2})(i\in {\rm{root}},\,j\in {\rm{branch}}\,{\rm{B}})\,,$$

for other weights. The value of ${w^{\prime} }_{\max }$ was 14. Initial synaptic weights from EC ${w}_{ij}^{{\rm{dnd}}}$ were uniformly sampled from the interval [0, 2].

The animal was immobile from 0 s to 60 s, and from 75 s to 135 s. In these periods, the number of firing sequences was counted in each branch to compare sequence propagation before and after an experience. During 60–75 s (first experience) and 135–150 s (second experience), *pos*(*t*) was changed in a similar way to the “first run” on the one-dimensional track (Fig. 3). The position *center*(*i*) of neuron *i* was uniformly sampled from [0, 1] for the first experience, and *center*(*i*) >0.2 were similarly resampled for the second experience. Trigger inputs were activated at the beginning of the first run, and the amplitude and length of each trigger input was 5 and 500 ms, respectively. We turned off synaptic plasticity during immobility to evaluate the effect of the previous experience explicitly. Other simulation settings were basically the same as in simulations of the one-dimensional track.

### Information per spike

We evaluated the accuracy of place fields by using information per spike given as follows:

71 $$\sum _{i}\frac{\lambda (po{s}_{i})}{\lambda }\,\mathrm{log}\,\frac{\lambda (po{s}_{i})}{\lambda }p(po{s}_{i}),$$

where *pos*_*i*_ is the binned position of the animal ($i=1,\ldots ,{N}_{{\rm{bin}}}$), *p*(*pos*_*i*_) is the probability that the animal is found at given position *i*, *λ* is the mean firing rate of the cell, *λ*(*pos*_*i*_) is the mean firing rate when the animal is in *pos*_*i*_. After removing immobile periods, we computed information per spike for all CA3 neurons having the mean firing rate higher than 1 Hz and averaged this quantity over these neurons. The number of bins *N*_bin_ was 50 in Figs 3 and 4; 75 (25 for one arm) in Fig. 6.

## Data Availability

All codes for simulations and visualization were written in Python 3 and available at https://github.com/TatsuyaHaga/preplaymodel_codes.
